# Characterization of Curtovirus V2 Protein, a Functional Homolog of Begomovirus V2

**DOI:** 10.3389/fpls.2020.00835

**Published:** 2020-06-19

**Authors:** Ana P. Luna, Beatriz Romero-Rodríguez, Tábata Rosas-Díaz, Laura Cerero, Edgar A. Rodríguez-Negrete, Araceli G. Castillo, Eduardo R. Bejarano

**Affiliations:** ^1^Departamento de Genética, Facultad de Ciencias, Instituto de Hortofruticultura Subtropical y Mediterránea “La Mayora” (IHSM-UMA-CSIC), Universidad de Málaga, Málaga, Spain; ^2^CONACyT, Departamento de Biotecnología Agrícola, Instituto Politécnico Nacional, CIIDIR-Unidad Sinaloa, Guasave, Mexico

**Keywords:** geminivirus, BCTV, RNA-silencing, suppressor, V2, RDR6, pathogenesis

## Abstract

Geminiviruses are single-stranded DNA plant viruses with circular genomes packaged within geminate particles. Among the *Geminiviridae* family, *Begomovirus* and *Curtovirus* comprise the two best characterized genera. Curtovirus and Old World begomovirus possess similar genome structures with six to seven open-reading frames (ORF). Among them, begomovirus and curtovirus V2 ORFs share the same location in the viral genome, encode proteins of similar size, but show extremely poor sequence homology between the genera. V2 from *Beet curly top virus* (BCTV), the model species for the *Curtovirus* genus, as it begomoviral counterpart, suppresses post-transcriptional gene silencing (PTGS) by impairing the RDR6/SGS3 pathway and localizes in the nucleus spanning from the perinuclear region to the cell periphery. By aminoacid sequence comparison we have identified that curtoviral and begomoviral V2 proteins shared two hydrophobic domains and a putative phosphorylation motif. These three domains are essential for BCTV V2 silencing suppression activity, for the proper nuclear localization of the protein and for systemic infection. The lack of suppression activity in the mutated versions of V2 is complemented by the impaired function of RDR6 in *Nicotiana benthamiana* but the ability of the viral mutants to produce a systemic infection is not recovered in gene silencing mutant backgrounds. We have also demonstrated that, as its begomoviral homolog, V2 from BCTV is able to induce systemic symptoms and necrosis associated with a hypersensitive response-like (HR-like) when expressed from Potato virus X vector in *N. benthamiana*, and that this pathogenicity activity does not dependent of its ability to supress PTGS.

## Introduction

Geminiviruses constitute a group of circular single-stranded DNA plant viruses that infect a wide range of plants and cause substantial yield losses worldwide (Rojas et al., 2018; García-Arenal and Zerbini, 2019). The family *Geminiviridae* is divided into nine genera based on their genome features and biological properties (Varsani et al., 2017; Zerbini et al., 2017). Among them, *Begomovirus* and *Curtovirus* include a large number of the viral species capable to infect economically relevant dicotyledonous plants. Curtoviruses are important pathogens for many cultivated and wild plant species. Although this genus only has three species, including the model species *Beet curly top virus* (BCTV), it has an enormously wide host range within dicot plants, including around 300 species in more than 40 plant families, as well as a broad geographical distribution that includes the Indian subcontinent, North and Central America and the Mediterranean region (Varsani et al., 2014).

All monopartite and Old World bipartite geminiviruses have similar genome structures, encoding 6 or 7 multifunctional proteins (Fondong, 2013; Varsani et al., 2017; Zerbini et al., 2017). In most cases, the virion sense strand contains two ORFs (V2 and V1, that encodes the coat protein, CP), and a third one (V3) which is only present in some of the nine genera, including *Curtovirus* but not *Begomovirus*. The complementary sense strand encompasses up to four ORFs (C1, C2, C3, and C4).

V2 from begomoviruses encodes a multifunctional protein required for full infection that is able to suppress gene silencing at the transcriptional (TGS) and post-transcriptional (PTGS) level (Zrachya et al., 2007; Chowda-Reddy et al., 2008; Sharma and Ikegami, 2010; Sharma et al., 2010; Amin et al., 2011; Luna et al., 2012; Zhang et al., 2012; Wang et al., 2014, 2018; Saeed et al., 2015; Zhan et al., 2018; Mubin et al., 2019). Additionally, it has been described that V2 protein is required for viral movement and spreading of the virus throughout the plant (Padidam et al., 1996; Rojas et al., 2001; Rothenstein et al., 2007; Moshe et al., 2015), it is involved in the regulation of host defense responses (Bar-Ziv et al., 2012; Roshan et al., 2018) and it elicits symptoms of hypersensitive response (HR)-like cell death phenotype in *Nicotiana benthamiana* plants, when expressed from a *Potato virus X* (PVX)-derived vector (Zrachya et al., 2007; Chowda-Reddy et al., 2008; Mubin et al., 2010; Amin et al., 2011; Luna et al., 2012; Zhang et al., 2012; Roshan et al., 2018).

Less is known about the function of curtovirus V2. Based on genome location and length, begomovirus and curtovirus V2 ORFs seem to be orthologous. However, their homology at the protein level, which is high within each genus, is extremely low. As its begomovirus counterpart, curtovirus V2 is needed for full systemic infection (Stanley et al., 1992; Hormuzdi and Bisaro, 1993; Luna et al., 2017) and it functions as a strong PTGS suppressor by a similar mechanism: impairing the RDR6 (RNA-dependent RNA polymerase 6)/suppressor of gene silencing 3 (SGS3) pathway (Luna et al., 2017).

Here we show that BCTV V2, besides functioning as a PTGS suppressor, also produces an HR-like cell death phenotype in *N. benthamiana* when expressed from PVX, similar to that produced by the expression of its begomoviral equivalent. The functional analysis of the mutated versions of V2 indicated that a putative phosphorylation motif and two N-terminal hydrophobic domains conserved also in V2 from begomovirus, are required for the PTGS suppression activity, viral pathogenicity, V2 subcellular localization and for systemic infection of *N. benthamiana* and *Arabidopsis thaliana* plants. Collectively, these results suggest that begomovirus and curtovirus V2 proteins, in spite of their low sequence homology, have evolved to target the same cellular processes through similar mechanisms, providing a putative example of convergent protein evolution.

## Materials and Methods

### Microorganisms, Plant Material, and Growth Conditions

Manipulation of *Escherichia coli* strains were carried out according to standard methods (Sambrook and Russell, 2001). *E. coli* strain DH5-α was used for subcloning. The *Agrobacterium tumefaciens* GV3101 strain was used for the agroinfiltration and agroinoculation/infection assays.

Plants used in this study were *A. thaliana* Columbia (Col-0) ecotype and *Nicotiana benthamiana*. Plants were grown in chambers at 24°C in long-day conditions (16 h light/8 h dark) before and after agroinfiltration/infection. The *Arabidopsis* mutant lines used for geminiviral infection, *rdr6–15* and the double mutant *dcl2/4*, were described elsewhere (Allen et al., 2004; Deleris et al., 2006, respectively). *N. benthamiana* RDR6i transgenic line was described in Schwach et al., 2005).

### Plasmids and Cloning

Supplementary Tables S1, S2 summarize the constructs and the primers used in this work, respectively. All PCR-amplified fragments cloned in this work were fully sequenced. The binary vector expressing green fluorescent protein (GFP) (pBin-35S-mGFP5) (Haseloff et al., 1997) and the nuclear envelope marker AtSUN1–tagRFP (Oda and Fukuda, 2011) were kindly provided by Olivier Voinnet (Zurich, Switzerland) and by Björn Krenz (Braunschweig, Germany), respectively. V2 single mutants, except V2P1D, were generated using two-sided splicing by over-lap extension (Ho et al., 1989). Primers used for both amplification rounds are shown in Supplementary Tables S2, S3. The V2P1D mutant and the BCTV V2 mutant viruses were produced by site-directed mutagenesis using QuikChange Lightning Site-Directed Mutagenesis kit (Stratagene, Agilent biotechnologies) with specific primers (see Supplementary Tables S2, S4).

### Analysis of Nucleic Acids and Proteins

Nucleic acid manipulation was performed according to standard methods (Sambrook and Russell, 2001). For BCTV replication and infection analyses, plant DNA was extracted from the infiltrated (local) or the apical (systemic) leaves of the infected plants at 4 or 28 days post-infiltration (dpi), respectively, digested with *Dpn*I to remove bacterial DNA in the infiltrated tissues (local infection) and then subjected to qPCR analysis using primers described on Supplementary Table S2 and the 25SrDNA and actin genes as normalizers for *N. benthamiana* and *A. thaliana* samples, respectively (Luna et al., 2017). Expression of BCTV V2 (wild-type or mutants) and GFP in agroinfiltrated tissues was determined by RT-qPCR using the primers described on Supplementary Table S2 and the E1Fa gene as a normalizer (Luna et al., 2017). The analysis of PVX transcript levels and recombinant virus integrity were done by semi quantitative RT-PCR as described in Luna et al., 2012 using specific primers (Supplementary Table S2).

For western blot analysis, 100 mg of leaf tissue per sample were used. Total protein was extracted with two volumes of extraction buffer (100 mM Tris–HCl pH 7.5, 150 mM NaCl, 10% glycerol, 0.5 mM EDTA pH 8, 1 mM DTT, 0.5 mM PMSF, 1%[v/v] P9599 protease inhibitor cocktail [Sigma-Aldrich]; 0.2%[v/v] triton X-100). Samples were centrifuged 15 min at 4°C at 16000 *g*. Approximately 150 μl of total protein was recovered, mixed with an equal volume of 2X Laemmli buffer and heated at 95°C for 10 min. 20 μl of total protein was loaded and resolved by 12% SDS-PAGE gel electrophoresis, and transferred by electroblotting onto a PVDF membrane (Immobilon-P, Millipore, MA, United States). Proteins were stained by Coomassie blue and immunoblotted with anti-GFP mouse monoclonal antibody (1:600, clone B-2; sc-9996, Santa Cruz Biotechnology) and anti-mouse IgG whole molecule-peroxidase secondary antibody (1:80,000; A9044, Sigma-Aldrich).

### Agroinfiltration and Infection Assays

For PTGS local suppression assays and PVX infections, *N. benthamiana* leaves were agroinfiltrated as previously described (Voinnet et al., 1998; Luna et al., 2012). Long-wave UV lamp was used for the detection of GFP fluorescence (Black Ray model B 100 AP, Upland, United States).

To quantify the hypersensitive-response (HR) in PVX-infected plants, conductivity (mS/cm) was measured using a conductivity meter Crison CM35 (Hach-Lange, Barcelona, Spain). Leaf discs were cut from infiltrated (local tissue) or apical leaves (systemic tissue), washed for 30 min in 6 mL of deionized water, and then transferred to 6 mL of deionized water and the conductivity was measured every 24 h for a total of 3 days, starting at 6 dpi (local tissue) or 8 dpi (systemic tissue). For BCTV infection in *A. thaliana* plants, 4-5-week-old plants were agroinoculated by needle puncture in wounds produced in the rosette crown. 2–3 drops of an overnight grown *A. tumefaciens* culture were placed over these wounds, plants were covered in plastic film for 2–3 days and then plastic was removed. BCTV infection in *N. benthamiana* was done by agroinoculation as described by Elmer et al., 1988.

### Subcellular Localization

For immunolocalization, *A. tumefaciens* was transformed with binary vectors containing V2 mutants fused to GFP. *N. benthamiana* leaves were agroinfiltrated with cultures at OD_600_ 0.5–1. For co-localization experiments Agrobacterium cultures containing V2-GFP constructs and the nuclear envelope marker AtSUN1–tagRFP (Oda and Fukuda, 2011) were mixed (1:1 ratio) prior to infiltration. In both cases, fluorescence was detected in epidermal cells 2–3 days after infiltration, using a confocal microscope (Zeiss LSM 880).

### Phylogenetic Analysis

The ClustalW algorithm was used to align V2 homolog proteins^1^. The prediction of the putative phosphorylation motives was done by Scan Prosite tool^2^. The hydrophobic domains were identified by ProtScale software^3^.

## Results

### A Phosphorylation Motif and Two Hydrophobic Domains of BCTV V2 Protein Are Essential for Its Silencing Suppression Activity

**FIGURE 1:**
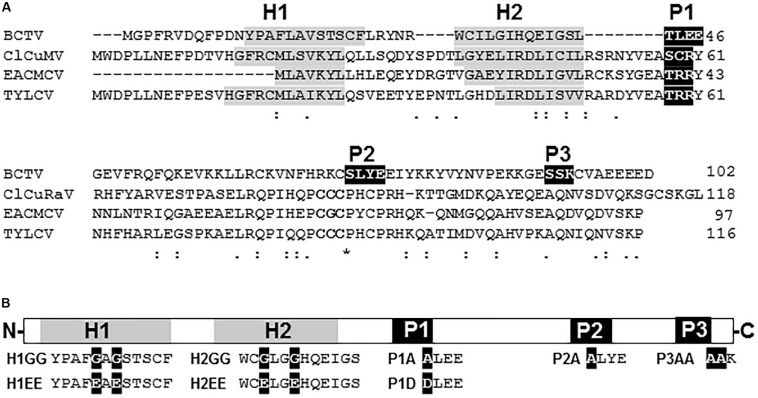
V2 sequence from curtovirus and begomovirus. **(A)** Alignment of the aminoacid sequences of the V2 proteins from the curtovirus *Beet curly top virus* (BCTV; M24597) and the begomoviruses, *Cotton leaf curl Multan virus* (CLCuMV-Fai[PK:Fai2]; AJ496287), *East African cassava mosaic Cameroon virus* (EACMCV; AF112354) and *Tomato yellow leaf curl virus* (TYLCV; X15656). Gaps (-) were introduced to optimize the alignment. The positions of the predicted putative phosphorylation motifs P1 (protein kinase CK2/protein kinase C), P2 (protein kinase CK2) and P3 (protein kinase C) are depicted in white letters inside black boxes. The CxC motif from begomoviruses (Padidam et al., 1996; Zrachya et al., 2007) is underlined. The hydrophobic domains (H1 and H2) are shadowed in gray. **(B)** Schematic view of BCTV V2 aminoacidic sequence. Hydrophobic domains (H1 and H2) are depicted as gray squares. Putative phosphorylation motifs (P1, P2 and P3) are presented as black squares. Amino acid substitutions for each mutant (H1GG, H1EE, H2GG, H2EE, P1A, P1D, P2A, and P3AA) are indicated as white letters in black squares.

Suppression of PTGS activity has been described for V2 proteins from several species of begomovirus and the curtovirus *Beet curly top virus* (BCTV) (see section “Introduction” for references). Despite sharing a similar function, the protein homology, which is highly conserved within each genus (Supplementary Figures S1, S2), is extremely poor when V2 from begomovirus and curtovirus are compared (Figure 1A). In begomoviral V2, two highly conserved protein domains have been identified to be essential for its activity as PTGS suppressor (Supplementary Figure S1): (i) a putative CK2/PKC (protein kinase CK2/protein kinase C) phosphorylation motif (Chowda-Reddy et al., 2008) and (ii) a CxC motif (Glick et al., 2008) that is also required for full infection (Hak et al., 2015). Sequence analysis revealed that V2 from curtoviruses lacks the CxC motif, but it contains several putative phosphorylation sites (Supplementary Figure S2). Sequence alignment showed that one CK2/PKC motif (hereafter named P1) is conserved in all curtoviral species (Supplementary Figure S2) and seems to be homologous to the begomoviral CK2/PKC motif (Figure 1 and Supplementary Figure S1). On the other hand, the other two putative CK2 and PKC phosphorilation motifs predicted in BCTV V2 (named P2 and P3, respectively), seemed to be specific, since they are not conserved in the other two curtoviral species neither in the begomoviral V2 (Figure 1 and Supplementary Figure S2). Interestingly, when hydrophobic profiles of begomoviral and curtoviral V2 proteins are compared, a similar disposition consisting of two hydrophobic domains in the N-terminal region (hereafter named H1 and H2) followed by a long hydrophilic domain in the C-terminus is revealed (Figure 1A). A broader alignment of V2 from 29 begomovirus and 3 curtovirus species, displayed a similar distribution of the hydrophobic-hydrophilic domains (Supplementary Figures S1, S2).

To determine the functional relevance of the hydrophobic domains and the phosphorylation motifs in the PTGS suppression activity of BCTV V2, we generated point mutants in the mentioned domains (Figure 1B and Supplementary Table S3). Non-polar residues from the hydrophobic domains, leucine and valine from H1 and two isoleucines from H2, were substituted by either polar-charged amino acids such as glutamic acid (H1EE and H2EE) or by a non-polar such as glycine (H1GG and H2GG). The serine/threonine residues in the three hypothetical phosphorylation motifs were replaced by alanines (P1A, P2A, and P3AA) or by aspartic acid in the case of P1 (P1D) to create a phosphomimic mutation. Finally, as a control, an insertion of two nucleotides (AT) at position 395 in BCTV genome, generated a premature stop signal that produced a peptide of 17 aminoacids whose first 5, correspond to V2 protein.

**FIGURE 2:**
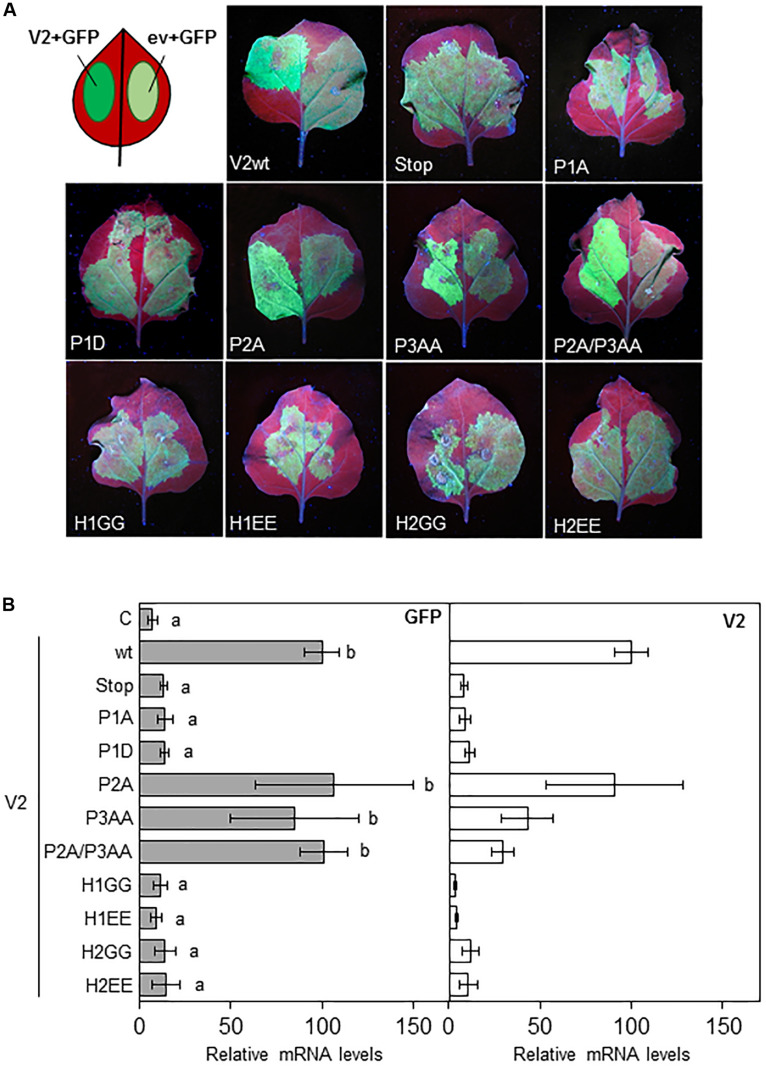
Local PTGS suppression activity of V2 mutants in wild-type *N. benthamiana* plants. **(A)** Leaves from *N. benthamiana* plants infiltrated with a mixture of two *A. tumefaciens* cultures expressing GFP and the indicated version of V2, were photographed under UV light at 5 dpi. Wild-type V2 protein (wt) and the empty vector (C) were used as a positive and negative control, respectively. **(B)** Relative *GFP* and *V2* mRNA levels (RT-qPCR) in infiltrated tissues at 5 dpi. *GFP* or *V2* transcript levels were normalized to *EF1* and are presented as the relative amount of transcripts compared with the amount found in wild-type V2 (wt) samples (set to 100%). Bars represent mean values ± standard error (SE) for 4–8 pools of two leaves from 3 to 4 plants each one. Mean values marked with different letter (a or b) indicate results significantly different from each other, as established by One Way ANOVA (Dunnett’s Multiple Comparison Test; *P* < 0.05).

To evaluate the gene silencing suppressor activity of the V2 mutants, *N. benthamiana* wild-type leaves were co-agroinfiltrated as described in Luna et al. (2012) with constructs that expressed GFP and the desired V2 mutant protein from the 35S CaMV promoter. As a negative control the 35S-GFP construct was also co-agroinfiltrated on each leaf with an empty binary vector (ev and C) (Figure 2A). As expected, 5 days after the infiltration (5 dpi) leaf patches agroinfiltrated with the 35S-GFP construct and wild-type V2, displayed stronger green fluorescence compared to those infiltrated with the empty vector and 35S-GFP (Figure 2A; Luna et al., 2017). A similar result was observed in tissues expressing P2A and P3AA, but not in tissues infiltrated with V2stop, P1A, P1D or any of the V2 mutants at the hydrophobic domains, where the GFP signal was similar to the one observed in the control (Figure 2A). To confirm that P2A and P3AA motifs are not required for the suppressor activity of V2, we generated a double mutant (P2A/P3AA) and analyzed its ability to suppress GFP silencing. The level of fluorescence at the leaf patches co-agroinfiltrated with 35S-GFP and the double mutant P2A/P3AA were similar to that obtained in tissues expressing wild-type V2 (Figure 2A). The ability of the V2 mutants to supress gene silencing was also determined by measuring the relative transcript levels of GFP by reverse transcription quantitative real-time PCR (RT-qPCR). Transcripts accumulated to a similar extend in the tissues expressing wild-type V2 or the single or double mutants P2A/P3AA and to a lesser extend in tissues agroinfiltrated with any of the other V2 mutants (Figure 2B). A similar result was obtained with the relative amount of V2 transcripts, indicating that when functional, V2 also suppresses its own silencing. To determine whether the low level of transcript accumulation of the V2 mutants was due to the lack of PTGS suppression activity and not to a reduction in the transcript generation, we measured the accumulation of V2 transcripts at 1 dpi, before gene silencing is stablished (Supplementary Figure S3). The results indicated that, although we detected some variation, the level of V2 transcript from the mutants and wild-type were similar and they do not correlate with their ability to suppress GFP expression, confirming that the differences observed at 5 dpi are due to the lack of gene silencing suppression activity of V2 mutants.

**FIGURE 3:**
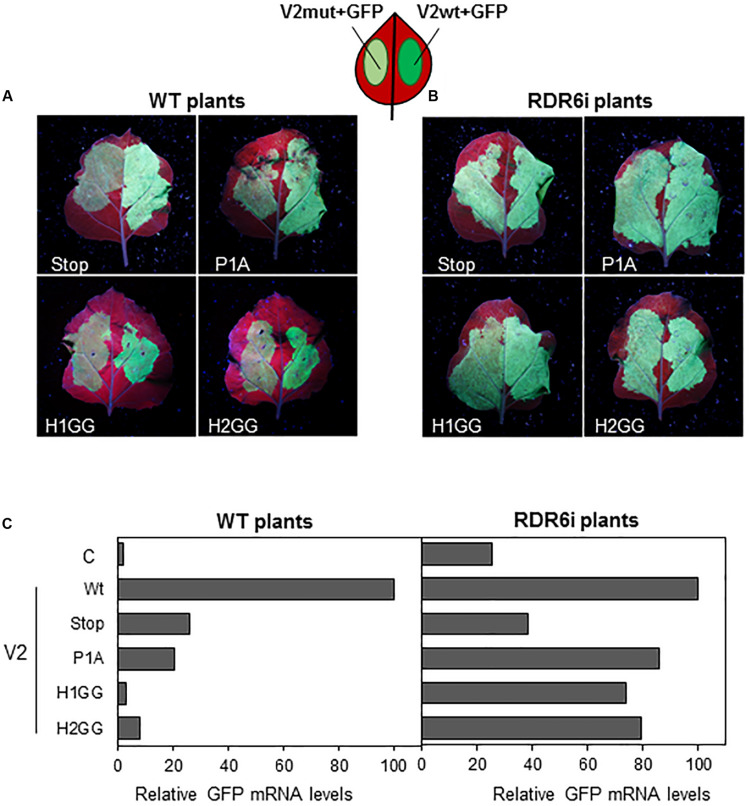
Local PTGS suppression activity of V2 mutants in wild-type (WT) and RDR6i *N. benthamiana* plants. Leaves from WT **(A)** and RDR6i **(B)** *N. benthamiana* plants infiltrated with a mixture of two *A. tumefaciens* cultures containing GFP and the desired V2 construct, were photographed under UV light at 5 dpi. Wild-type V2 protein (wt) was used as a positive control. **(C)** Relative GFP mRNA levels in infiltrated tissues at 5 dpi. GFP levels were normalized to *EF1* and are presented as the relative amount of transcripts compared with the amount found in wild-type V2 (wt) samples (set to 100%). Bars represent mean values from a pool of 2–3 leaves obtained from 4 plants for each combination. Similar results were obtained in two independent experiments.

Previous data suggested that, in *Arabidopsis thaliana*, V2 from BCTV suppresses PTGS by interfering with the RDR6-dependent amplification pathway (Luna et al., 2017). To confirm that this pathway was also required for the gene silencing suppression mechanism in *N. benthamiana*, we took advantage of the RDR6i line (Schwach et al., 2005), in which *NbRDR6* expression is reduced by an RNAi hairpin construct. Leaves from wild-type and RDR6i plants were co-infiltrated with two Agrobacterium cultures harboring plasmids to express GFP and the wild-type or the mutated V2 (only one mutant for each domain, P1A, H1GG and H2GG, were included in the analysis as the two type of mutations on each domain, were unable to suppress gene silencing) (Figure 2). As expected, leaf patches agroinfiltrated with wild-type V2 showed stronger green fluorescence compared to tissues co-infiltrated with any of the four mutants in *N. benthamiana* wild-type plants (Figure 3A). On the other hand, the green fluorescence signal of the patches that expressed the mutated versions of V2 in the RDR6i background was similar to that shown by wild-type V2 (Figure 3B). The relative quantification of GFP transcripts accumulated in the infiltrated tissues, confirmed these observations, as the increase of GFP transcript levels in P1A, H1GG and H2GG mutants reached similar levels to the ones from the V2 wild-type protein in the RDR6i background (Figure 3C), indicating that the lack of silencing suppression activity of V2 mutants, is complemented by the impaired RDR6 function in the RDR6i line. These results support the view that BCTV V2-mediated silencing suppression operates via hindrance of the RDR6 function in *N. benthamiana*, and it is in accordance with our previous data from *A. thaliana* (Luna et al., 2017).

### Pathogenicity of BCTV V2 Does Not Dependent of Its Ability to Supress PTGS

**FIGURE 4:**
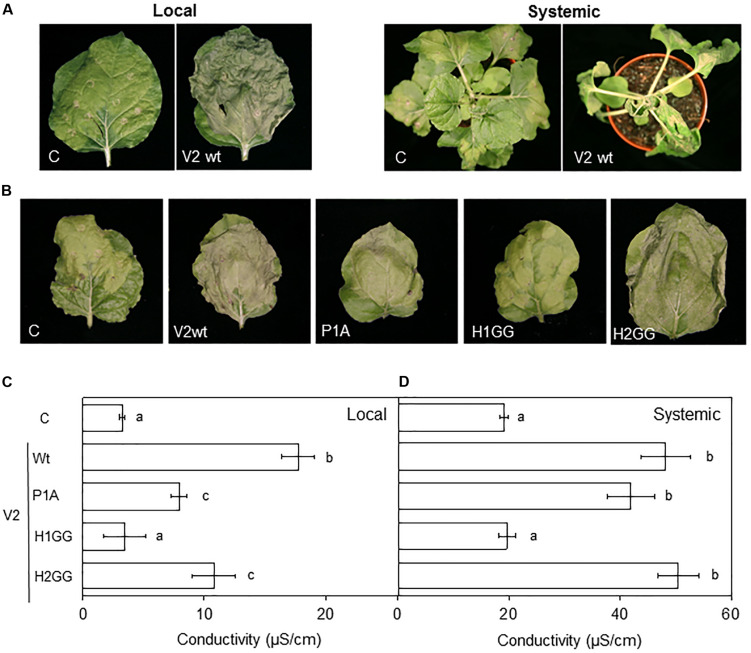
Expression of BCTV V2 mutants from PVX. Wild-type or mutated versions of V2 were expressed from a PVX vector and tested individually in *N. benthamiana* leaves. **(A)** Infiltrated tissue (local) at 6 dpi or apical tissue at 10 dpi (systemic) with the empty PVX vector (C) or with a recombinant PVX virus expressing the wild-type V2 protein (V2wt). **(B)** Infiltrated tissue (local) at 6 dpi with the empty PVX vector (C) or with a recombinant PVX virus expressing the wild-type (V2wt) or the mutated versions (P1A, H1GG, H2GG) of V2. **(C,D)** Measurement of the conductivity from leaf discs extracted from local **(C)** or systemically infected **(D)** tissues. Seven to eight plants were infected with each PVX-derived construct and conductivity (μS/cm) was measured in two pools of four leaf discs per plant. Bars represent mean values ± standard error (SE) for seven to eight plants (four discs per leaf were pooled and two leaves per plant were used) per recombinant PVX construct. Mean values marked with different letter (a, b, or c) indicate results significantly different from each other, as established by One Way ANOVA (Dunnett’s Multiple Comparison Test; *P* < 0.05). Similar results were obtained in two independent experiments.

Previous results indicated that begomoviral V2 is involved in pathogenicity as the ectopic expression in *N. benthamiana* of V2 from several begomoviruses using a *Potato virus X* (PVX)-derived vector, caused localized cell death in the infiltrated area and induced systemic symptoms and necrosis associated with a hypersensitive response-like (HR-like) phenotype (Mubin et al., 2010; Sharma and Ikegami, 2010; Luna et al., 2012; Roshan et al., 2018). Although stable expression of BCTV V2 in transgenic *A. thaliana* Col-0 plants do not alter the plant phenotype (Luna et al., 2017), we studied whether the curtoviral protein, as its begomoviral equivalent, induces symptoms and local or systemic necrosis, when it is expressed from a PVX-derived vector. Tissues infiltrated with the PVX empty vector (control, C) developed the typical local yellowing symptoms but the presence of V2 produced a HR-like cell death phenotype in the infiltrated *N. benthamiana* leaves (Figure 4A, local panels). At a systemic level, PVX infection (control, C) produced mild mosaic symptoms that came to be asymptomatic in some leaves due to recovery from viral infection. On the other hand, when BCTV V2 was expressed from PVX, plants did not recuperate from viral infection, collapsing at 8–10 dpi (Figure 4A, systemic panels). Therefore, we can conclude that as its begomoviral counterpart, V2 from curtovirus induces an HR-like phenotype in *N. benthamiana* when is expressed from PVX.

In order to determine the relevance that the selected protein domains have in V2 pathogenicity and if that function is related to the gene silencing suppressor activity, we expressed the curtoviral V2 mutant proteins (P1A, H1GG, and H2GG) from a PVX-derived vector in *N. benthamiana* plants. Local and systemic necrosis was observed in samples expressing wild-type V2 and the P1A and H2GG V2 mutants but not the H1GG mutant (Figure 4B and data not shown). To quantify the HR caused by wild-type and mutants in V2, we measured the changes in the conductivity of the water that are produced by the release of cellular electrolytes in the presence of a pathogenic factor (Baker et al., 1991; Mackey et al., 2002). Conductivity of the leaf discs extracted from the local (6dpi) or systemically infected (9 dpi) tissues was measured. In the infiltrated tissue (local infection), the maximum electrolyte leakage was elicited by the V2 wild-type protein and the initiation of the response reaction seemed to be delayed in P1A and H2GG mutants (Figure 4C). In apical tissue (systemic infection) the V2 wild-type protein and the P1A and H2GG mutants elicited a similar HR in terms of electrolyte leakage (Figure 4D). As previously observed by symptoms (Figure 4B), there were not significant differences between the conductivity levels detected in the H1GG mutant expressed from PVX and PVX, either locally or systemically (Figures 4C,D, respectively).

Symptoms intensification produced by PVX-recombinant viruses expressing other viral proteins has been related to a larger accumulation of genomic RNA from PVX (Cañizares et al., 2008; Zhang et al., 2012). To rule out this possibility, total RNA was obtained from the systemically infected tissue and PVX was detected by RT-PCR. We did not detect an increase in PVX genomic RNA accumulation with respect to the control samples (Supplementary Figure S4). Amplification using primers flanking the PVX vector cloning site, confirmed the integrity of V2 sequences in the recombinant viruses (Supplementary Figure S4).

### Mutations in P1 or H2 Domains Alter the Subcellular Localization of V2 Protein

**FIGURE 5:**
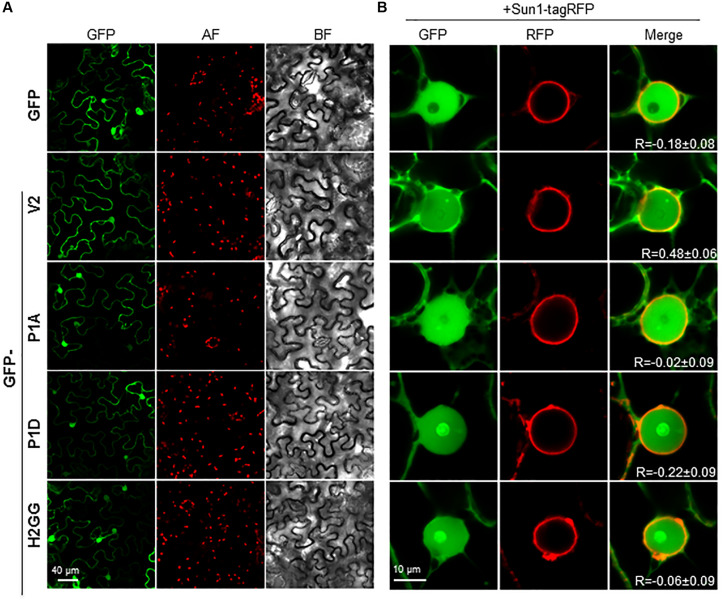
Subcellular localization of V2 mutants fused to GFP protein in epidermal cells of *N. benthamiana.* **(A)** Leaves were agroinfiltrated with a construct expressing the 35S:GFP (GFP), 35S:GFP-V2 fusion protein or the 35S:GFP-V2 mutants. Samples were observed under the confocal microscope at 2 dpi. GFP fluorescence (GFP), autofluorescence (AF) and the bright field channel (BF) are shown. **(B)** Co-localization of 35S:GFP-V2 (wt and mutants) and the nuclear envelope marker 35S:AtSUN1–tagRFP. Leaves were agroinfiltrated with a construct expressing the GFP-V2 fusion protein or the GFP-V2 mutants and the nuclear envelope marker AtSUN1–tagRFP and observed under the confocal microscope at 3 dpi. GFP fluorescence (GFP), RFP fluorescence (RFP), and merge are shown. R stands for the Pearson’s correlation coefficient for GFP-fusions and SUN1-tagRFP (mean values ± standard error (SE) from 6 to 9 nuclei).

We have previously shown that BCTV V2 localizes in the ER network and in the nucleus, from the perinuclear region to the cell periphery (Luna et al., 2017). We addressed whether mutations in the H1, H2 or P1 domains could change the subcellular localization of the protein by transiently expressing GFP-fused versions of BCTV V2 in *N. benthamiana*. Leaves were agroinfiltrated, collected 2–3 days later and visualized using a confocal microscope. Wild-type GFP-V2 localized in the nucleus and in the cellular periphery and not significant differences were observed in the localization of the mutated V2 proteins (Figure 5A). However, a close-up of the images showed some differences between the nuclear localization of wild-type V2 and the mutants, as GFP-P1A, GFP-P1D and GFP-H2GG, lose their nuclear periphery localization. To confirm this observation, we transitorily co-expressed the different GFP-V2 proteins with the nuclear envelope marker AtSUN1 tagged with RFP (Oda and Fukuda, 2011). Upon co-expression, overlapping of the fluorescent signals from GFP and RFP was detected for wild-type GFP-V2 protein but not for GFP-H2GG, GFP-P1A, or GFP-P1D mutants, which showed similar values for the Pearson’s correlation coefficient to GFP and SUN1-RFP (Figure 5B and Supplementary Figure S5). Moreover, the GFP-V2 protein accumulated to a greater extend in the nucleolus in GFP-P1D but not in the GFP-P1A mutant, suggesting that the phosphorylation status of the P1 domain can play a role in the nuclear localization of BCTV V2 (Figure 5B). The GFP-H2GG mutant also accumulated in the nucleolus to a greater extent than the wild-type protein, indicating the importance of this hydrophobic domain for the proper localization of V2 protein. We could not determine the significance of the H1 domain as we were not able to detect the GFP-fused mutant protein (Supplementary Figure S6). It has been shown that in addition to the nucleoplasm, nuclear periphery and cytoplasm, V2 from the begomovirus TYLCV localizes in the Cajal body, upon transient expression in *N. benthamiana* (Wang et al., 2019, BioRxiv). Interestingly, we could observe that the wild-type and the mutated versions of V2, also localize in a discrete subnuclear compartment, that resembles the Cajal body (Figure 5B and data not shown) suggesting that BCTV V2 shares the same subcellular localization as its begomoviral equivalent.

### The Hydrophobic Domains H1 and H2 and the P1 Phosphorylation Motif of V2 Protein Are Essential for Systemic but Not for Local Infection of BCTV

**FIGURE 6:**
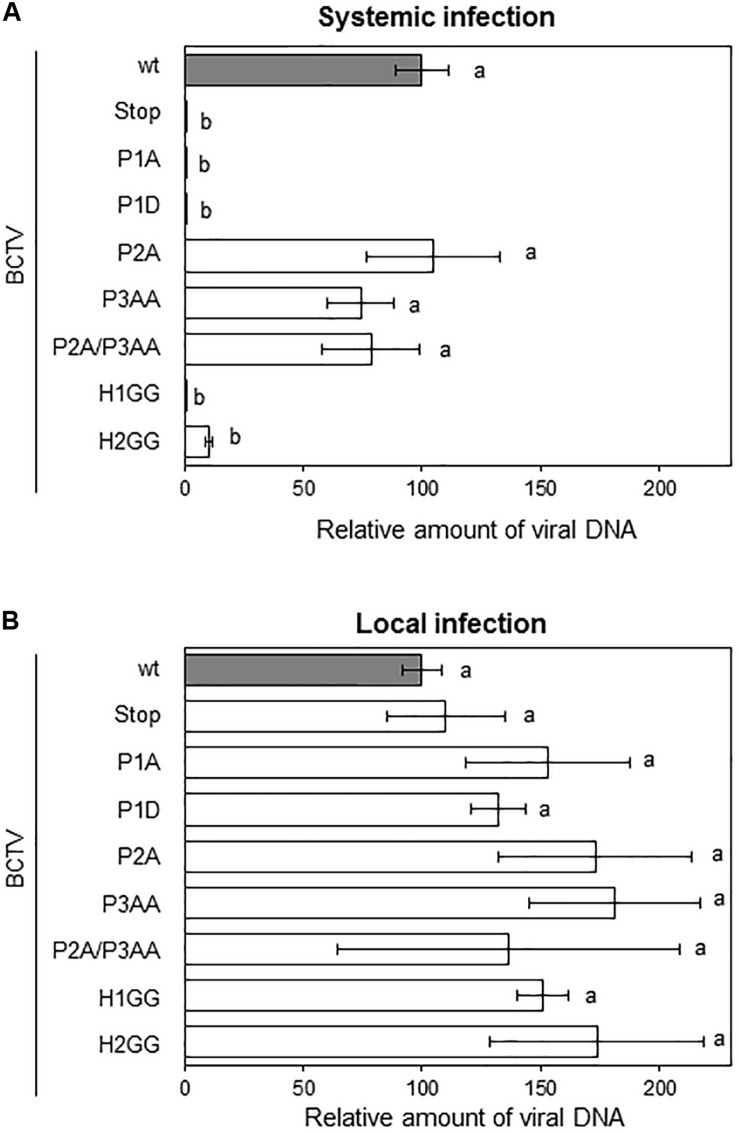
Infection of *N. benthamiana* plants with BCTV V2 mutants. Plants were agroinoculated with wild-type or V2 mutated BCTV infective clones. Relative viral DNA accumulation in apical leaves (systemic infection) at 28 dpi **(A)** or at the infiltrated leaves (local infection) at 4 dpi **(B)**. BCTV accumulation was measured by qPCR. DNA levels were normalized to 25S ribosomal DNA interspacer (ITS) and are presented as the relative amount of virus compared with the amount found in wild-type BCTV (wt) samples (in gray, set to 100%). Bars represent mean values ± standard error (SE) for 4–8 pools of two leaves from 3 to 4 plants each one. Mean values marked with different letter (a or b) indicate results significantly different from each other, as established by One Way ANOVA (Dunnett’s Multiple Comparison Test; *P* < 0.05).

To determine the biological relevance of V2 domains, we infected *N. benthamiana* with BCTV wild-type or BCTV mutated in the V2 hydrophobic or phosphorylation motifs. Plants inoculated with wild-type virus developed typical symptoms of BCTV infection by 14 to 21 dpi. Viruses containing single or the double mutations in P2 and P3 domains, caused symptoms indistinguishable from those produced by the wild-type virus, indicating that these mutations do not affect the systemic infection. In contrast, plants inoculated with the virus containing a mutation in the hydrophobic domain H2 (H2GG) developed only mild leaf curling and chlorosis. Viruses containing the premature stop codon in V2 (V2stop), the mutations in the P1 phosphorylation motif (P1A and P1D) or in the hydrophobic domain H1 (H1GG) did not produce any detectable symptoms at 28 dpi (Supplementary Figure S7). To quantify BCTV DNA levels in infected *N. benthamiana* plants, samples from apical leaves were analyzed by qPCR at 28 dpi. Accumulation of viral DNA correlated with symptoms intensity. Plants infected with wild-type or P2A, P3AA, or P2A/P3AA mutant viruses, accumulated equivalent amounts of viral DNA (Figure 6A). A reduction in viral DNA levels was detected in plants infected with the H2GG mutant, suggesting that mild symptoms are associated with lower amount of viral DNA. No viral DNA was observed in any of the plants infected with the P1A, P1D, H1GG or V2stop mutants (Figure 6A). We confirmed by sequencing the V2 ORF, that in plants infected with the P2A, P3AA, P2A/P3AA, or H2GG mutants the viral DNA did not result from the replication of revertants, as all the analyzed fragments contained the proper mutation, confirming that these mutations are stable in infected plants.

To discern if the lessen or absent viral DNA levels in plants infected with the mutants was due to a defect in replication and/or spreading throughout the plant, we quantified the viral DNA in the agroinfiltrated leaves at 4 dpi (local infection, from the same *N. benthamiana* plants used in the systemic infection assay, Figure 6A). DNA was extracted and analyzed by qPCR. As presented in Figure 6B, all the mutants supported viral replication, indicating that the total or partial inability of P1A, P1D, H1GG, H2GG, and V2stop mutant viruses to infect *N. benthamiana* plants was not due to a replication defect. These findings suggested that the impairment of the BCTV V2 mutants to systemically infect the plant is related with the movement and dissemination of the viruses through the plant tissues.

**FIGURE 7:**
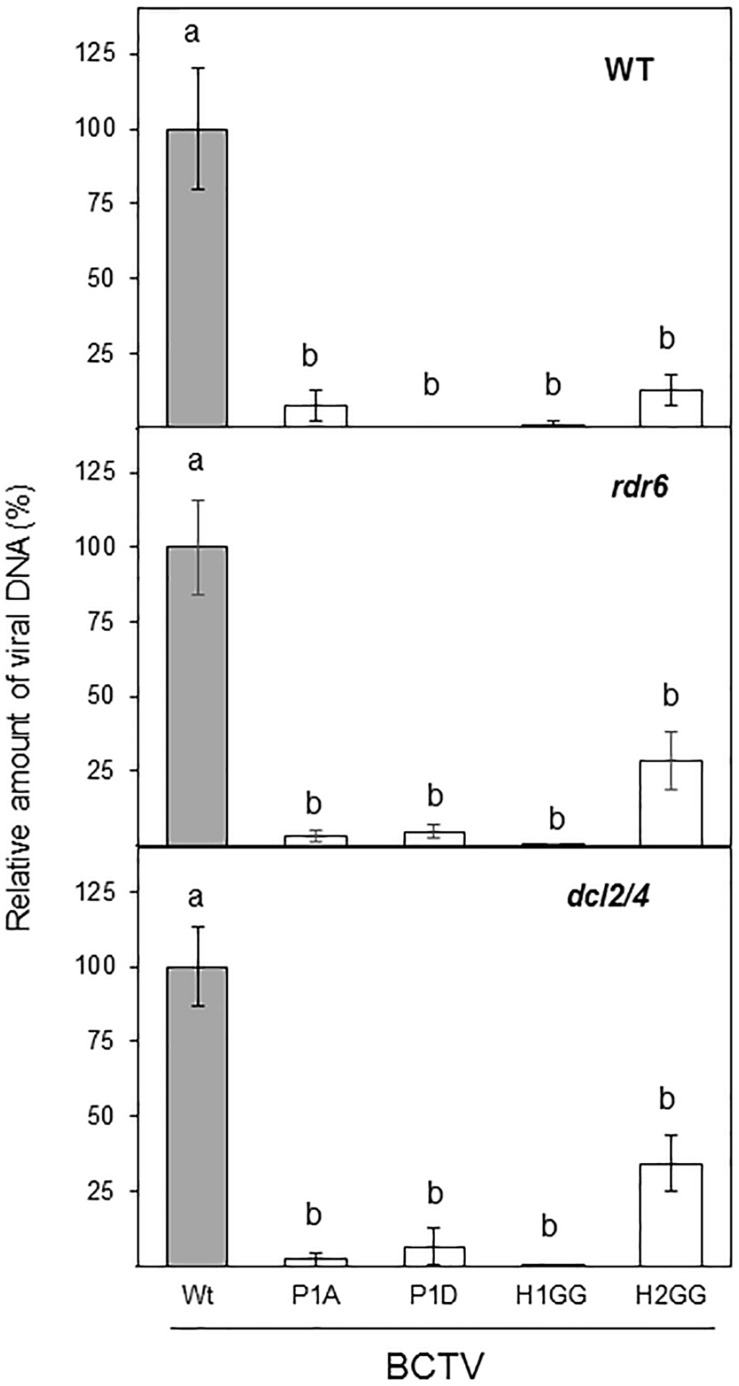
Infection of *A. thaliana* wild-type (WT), *dcl2/4* (*dcl2-1/dcl4-2*) and *rdr6 (rdr6-15)* mutants with wild-type or BCTV V2 mutants. The relative viral DNA accumulation at 28 dpi was quantified by qPCR in WT, *rdr6* and *dcl2/4* backgrounds. DNA levels were normalized to actin gene and are presented as the relative amount of virus compared with the amount found in wild-type BCTV (wt) samples (in gray, set to 100%). Bars represent mean values ± standard error (SE) for eight to twelve plants per biological replicate. Data from three biological replicate are shown. Mean values marked with different letter (a or b) indicate results significantly different from each other, as established by One Way ANOVA (Dunnett’s Multiple Comparison Test; *P* < 0.05).

We have previously demonstrated that V2 from BCTV suppresses PTGS by interfering with the RDR6-dependent amplification pathway in *A. thaliana* (Luna et al., 2017). Our data indicate that the lack of silencing suppression activity of the V2 mutants P1A, H1GG and H2GG, is complemented by the impaired function of RDR6 in the *N. benthamiana* RDR6i line (Figure 3). As an additional approach to analyze whether the lack of these components of the antiviral silencing pathway, could genetically complement the defective systemic infection of BCTV V2 mutants, we infected *A. thaliana* mutants deficient in RDR6 (*rdr6-15*) and DCL2 and DCL4 (double mutant *dcl2-1/dcl4-2* or *dcl2/4*) with BCTV wild-type and V2 mutants unable to systemically infect *N. benthamiana*. *A. thaliana* Col-0 plants infected with BCTV were clearly symptomatic, whereas plants infected with any of the BCTV mutants did not develop symptoms (data not shown). The qPCR results from the infected plants indicated that, as it occurs in *N. benthamiana*, mutant virus in P1 phosphorylation site (P1A and P1D) or any of the two hydrophobic domains (H1GG and H2GG) were impaired in systemic infection of *A. thaliana* (Figure 7). The differences on viral DNA accumulation among the wild-type and viral mutants were maintained when *rdr6-15* or the *dcl2/4* mutant plants were infected (Figure 7), indicating that the deficiency in viral infection caused by mutations in P1, H1, or H2 domains of V2, is not complemented by the impairment of the antiviral silencing pathway.

## Discussion

In this work, we have identified and characterized three domains of V2 from curtovirus required for PTGS suppression activity, subcellular localization and systemic, but not local infection. These results indicate that curtoviral V2 is, as its functional homolog the begomoviral V2, required for viral spreading in the plant.

The highly conserved protein kinase C (PKC) motif (P1) is required for the PTGS suppression activity of V2, as the replacement of the threonine residues of this motif impairs V2 ability to suppress GFP gene silencing in *N. benthamiana* leaves. The loss of PTGS suppression activity of P1A mutant is completely recovered in a RDR6-defective background, indicating that BCTV V2 suppresses PTGS through a mechanism that involves the RDR6-mediated pathway (Luna et al., 2017). However, the lack of suppression activity produced by the P1A mutation does not seem to be the cause of the lack on infectivity of this mutant, since infectivity is not even partially recovered in plants impaired in gene silencing (*rdr6* and *dcl2/4* mutants). Similar phenotypes are produced by the phosphomimic mutation of the P1 domain (P1D) suggesting that constitutive phosphorylation of the threonine 43 residue is not recovering the suppression activity, neither the ability to propagate the virus during the infection. In Old World begomovirus, this motif has been described to be involved in viral pathogenicity (Chowda-Reddy et al., 2008; Mubin et al., 2010) but studies have not been carried out to determine whether the domain is needed for PTGS suppression or systemic infection.

The analysis of V2 protein sequences drove us to study two hydrophobic domains (H1 and H2) located in the N-terminus, that are presented in all geminiviral V2 proteins. Replacement of two of the non-polar residues of those domains, dramatically affect the PTGS suppression activity of V2 and, as in the case of the P1 mutation, the activity is fully recovered when RDR6 activity is impaired. When *N. benthamiana* wild-type plants were infected with BCTV containing the H1GG or the H2GG V2 mutations, systemic infection was compromised although some differences were observed. While in wild-type *N. benthamiana* H1GG was fully impaired for systemic infection to a similar level than the null V2 mutant (V2stop), the virus containing the H2GG mutation showed a slightly more, although not statistically significant, viral DNA accumulation. This difference among both mutated viruses was more noticeable when *A. thaliana* plants impaired in gene silencing were infected. In wild-type plants infected with the H2GG mutant, the amount of viral DNA is only 12.6 ± 5.1% of the viral DNA accumulated in plants infected with the wild-type virus. However, in *rdr6* and *dcl2/4* mutants the quantity of H2GG viral DNA represented 28.3 ± 9.6% and 34.3 ± 9.2%, respectively. This indicated that the failure of H2GG to produce a systemic infection is partially recovered by the inhibition of PTGS antiviral activity. Altogether, these results indicated that the inability of these mutants to systemically infect the plant is not due to the lack of PTGS suppression activity. Therefore, other V2 function required for the viral movement is altered by those mutations. Interestingly, mutations in these domains, also provoked changes in the subcellular localization of V2 protein. As begomoviral V2, BCTV V2 protein localizes in the nucleoplasm and in the cell periphery, mainly associated to ER. Additionally, accumulation in the Cajal body (Guo et al., 2015; Wang et al., 2019, BioRxiv) and nuclear periphery (Zhou et al., 2011; Hak et al., 2015; Zhao et al., 2019, BioRxiv) have been reported for V2 from the begomoviruses TYLCV and Grapevine red blotch-associated virus. Here we describe that curtoviral V2 also accumulates in the nuclear periphery and in a discrete nuclear structure that could correspond to the Cajal body. Mutations in the P1 and H2 domains prevent the localization of the protein in the nuclear edge (Figure 5) but do not affect the accumulation in this nuclear body (data not shown). Begomoviral V2 interacts with the viral CP, a protein that seems to function as a nuclear shuttle protein that mediates nuclear import and export of viral DNA similarly to BV1, a protein encoded by the B-component of bipartite begomovirus (Rojas et al., 2001; Moshe et al., 2015; Zhao et al., 2019). In begomovirus, V2 affects the nuclear localization of CP and it enhances CP capacity to mediate nuclear export by a mechanism that depends on V2-CP interaction (Zhao et al., 2019). Mutation of cysteine residue (C85A) placed at the “x” position of the CxC motif from TYLCV V2, abolishes the interaction between CP and V2 and affects the nuclear export function and the perinuclear localization of V2. This mutation also causes delayed onset of very mild symptoms, indicating that the interaction between CP and V2 and, as a consequence, the V2-mediated nuclear export of CP is essential for viral spread in the plant. Curiously, our mutants in P1 and H2 displayed a surprisingly similar phenotype. Although curtoviral V2 lacks of a CxC motif, there is a cysteine residue (C71) conserved at a similar position. Considering the functional and structural similarities between curtoviral and begomoviral V2 proteins, a tantalizing possibility is that the BCTV V2 would also participate or mediate the nuclear export of the virus. This function could explain the impairment on viral systemic movement detected in BCTV V2 mutants. Further experiments to determine whether BCTV V2 interacts with CP and if this interaction is affected in the mutants, are in progress.

Hypersensitive response (HR) is an active defense reaction derived from the activation of defense-related pathways, which lead to cell death (CD) (reviewed in Heath, 2000). HR, which is characterized by rapid and localized cell death at the site of infection, arises after the interaction with an incompatible pathogen. Our results indicate that BCTV V2 functions as a pathogenicity determinant and possibly as an avirulence factor. Similar to the results reported for begomoviral V2, ectopic expression of BCTV V2 “*via”* a PVX-derived vector provoked the induction of local and systemic necrosis in the *N. benthamiana* leaves. Interestingly, BCTV infection in *A. thaliana* or *N. benthamiana* plants does not produce cell-death phenotype on systemically infected plants, maybe due to the limited number of cells that harbor its infection. Transient or permanent expression of V2 from a 35S CaMV promoter in *N. benthamiana* or *A. thaliana*, should increase the number of cells expressing V2 and bypass the tissue specificity. However, in none of the occasions in which BCTV V2 was expressed from those type of constructs a necrotic reaction was observed (Figure 2; Luna et al., 2017). This raises the concern of different levels of V2 protein accumulation in systemically infected tissues when PVX is used as the vector to express this curtoviral protein.

Interestingly, V2-induced HR is incapable to limit the long-distance movement of PVX-V2 resulting in systemic plant death (Figure 4). This function of BCTV V2 as pathogenicity determinant is similar to that described for V2 of Old World begomoviruses. However, the protein domains involved in this activity appear to be at least partially different. In begomovirus, deletion analysis and site-directed mutagenesis of the sequences encompassing the PKC phosphorylation motif (P1) have been shown to abolish or reduce the viral pathogenicity (induction of virus-like symptoms) and the ability to initiate HR (Chowda-Reddy et al., 2008; Mubin et al., 2010). Nevertheless, the mutation of the P1A or H2GG motifs of BCTV V2 reduced, but not abolished local, and did not affect systemic necrosis compared to the wild-type V2. This result suggests that the P1 motif and the H2 region of curtovirus are not fully required for the V2 to function as a pathogenicity determinant. On the contrary, expression of H1GG mutant displayed the same phenotype as the plants infected with non-recombinant PVX. Although this suggests that the H1 domain is absolutely required for the V2-induced HR, this result has to be taken cautiously, since we have not been able to detect a consistent expression of GFP-V2 fused protein containing the H1GG mutation. We cannot discard the possibility that the absence of HR induction by H1GG mutants is due to a lower protein level, in spite of that no difference with the wild-type V2 were observed at a transcriptional level (Supplementary Figure S3). Unfortunately, we do not have the antibodies against BCTV-V2 required to determine the protein level of V2H1GG. Although we cannot explain yet how V2 induces a response defense, our results clearly indicate that the ability of curtoviral V2 to induce HR is not dependent of its activity as PTGS suppressor since the PVX-derived expression of P1A and H2GG V2 mutants, that are heavily impaired in their suppression activity, showed similar values for conductivity and similar HR-like phenotype to the wild-type V2 (Figure 4).

The results presented here, confirm that V2 proteins from curtovirus and begomovirus are functional homologs. In spite of a very low level of sequence homology, they both are required for viral movement, present similar subcellular distribution and possess similar functions (PTGS suppressor and pathogenicity determinant). The fact that V2 ORF is present in Old World begomovirus and curtovirus, could suggest that this gene was already present in a common ancestor of both viral genera. However, the high level of conservation at the protein level among the species of each genus, but not between genera, and the absence of ORF V2 homologs in the genome of New World begomoviruses, argue against this hypothesis and suggest that V2 from curtovirus and begomovirus could have evolved independently.

## Data Availability Statement

All datasets generated for this study are included in the article/Supplementary Material.

## Author Contributions

AL, BR-R, and TR-D have performed the experiments, analyzed the data, and participated in the writing and critical reading of the manuscript. LC have provided technical assistance in the experimental procedures. ER-N has collaborated in some experiments and participated in the critical reading of the manuscript. EB and AC have planned and directed the experimental design of the work, have done the writing and the critical lecture of the manuscript. All authors contributed to the article and approved the submitted version.

## Conflict of Interest

The authors declare that the research was conducted in the absence of any commercial or financial relationships that could be construed as a potential conflict of interest.

## Footnotes

1 www.genebee.msu.su/clustal/advanced.html

2 https://prosite.expasy.org/scanprosite/

3 https://web.expasy.org/protscale/

## Supplementary Material

FIGURE S1 Alignment of the aminoacidic sequence of V2 from 29 begomovirus species: *Ageratum yellow vein virus-China* (AYVV/Gx; AJ495813), *Tomato yellow leaf curl Sardinia virus* (TYLCSaV; L27708), *Papaya leaf curl Guandong virus* (PaLCuGdV; AJ558122), *Indian cassava mosaic virus* (ICMV; AJ314739), *Cotton leaf curl Gezira virus* (CLCuGeV/Ca; AJ542539.1), *Honeysuckle yellow vein virus*-[UK] (HYVV-UK; AJ543429), *Tomato leaf curl virus-*Australia (ToLCV/To; S53251), *East African cassava mosaic Zanzibar virus* (EACMZV; AJ717559): *Tomato leaf curl Mayotte virus* (ToLCYTV; AJ620912), East African cassava mosaic Cameroon virus (EACMCV; AF112354); *Hibiscus rosa-sinensis mosaic virus)* (HRSMV; DQ022659), *Tomato leaf curl Laos virus* (ToLCLV; AF195782), *Tomato leaf curl Java virus* (ToLCJV; AB100304), *Tomato leaf curl China virus* (ToLCCNV; AJ811910), *Tomato leaf curl Taiwan virus* (ToLCTWV; DQ866127), *Spilanthes yellow vein virus* (SpYVV; DQ641694), *Sida yellow vein Vietnam virus* (SiYVVV; DQ641696), *Alternanthera yellow vein virus* (AlYVV; AM691015), *Tomato leaf curl Bangalore virus* (ToLCBV; AY428770), *Lindernia anagallis yellow vein virus* (LaYVV; AY795900), *Siegesbeckia yellow vein virus* (SgYVV; AM183224), *Tomato leaf curl New Delhi virus* (ToLCNDV; AY286316), *Tomato yellow leaf curl Indonesia virus* (TYLCIDV; AF189018), *Tomato leaf curl Hsinchu virus* (ToLCV; DQ866131), *Senecio yellow mosaic virus* (SeYMV; AJ876550), *Vernonia yellow vein virus* (VeYVV; AM182232), *Cotton leaf curl Multan virus* (CLCuMV; EF465535), *Ludwigia yellow vein virus* (LuYVV; AJ965539) and *Clerodendron yellow mosaic virus* (ClYMV; EF408037). Gaps (-) were introduced to optimize the alignment. The positions of the predicted putative phosphorilation motif P1 (protein kinase CK2/protein kinase C) are depicted in white letters inside black boxes. The CxC motif (Padidam et al., 1996; Zrachya et al., 2007) is underlined. The hydrophobic domains (H1 and H2) are shadowed in gray.

FIGURE S2 Alignment of the aminoacidic sequence of V2 from three curtovirus species: *Beet curly top virus-*California [Logan] (BCTV/CA/Logan; M24597), *Spinach severe curly top virus* (SpSCTV; GU734126) and *Horseradish curly top virus* (HCTV; U49907). The positions of the predicted putative phosphorylation motifs P1 (protein kinase CK2/protein kinase C), P2 (protein kinase CK2) and P3 (protein kinase C) are depicted in white letters inside black boxes. The hydrophobic domains (H1 and H2) are shadowed in gray.

FIGURE S3 Relative *V2* mRNA levels in *N. benthamiana* leaves. Leaves from *N. benthamiana* plants were infiltrated with a mixture of two *A. tumefaciens* cultures expressing GFP and the indicated version of V2 and relative *V2* mRNA levels were measured by RT-qPCR in the infiltrated tissues at 1 dpi. Wild-type V2 protein (wt) and the empty vector (C) were used as a positive and negative controls, respectively. *V2* transcript levels were normalized to *EF1* and are presented as the relative amount of transcripts compared with the amount found in wild-type V2 (wt) samples (set to 100%). Bars represent the mean ± SD for three different pools from 2 to 3 leaves obtained from 3–4 plants each one. One Way ANOVA (Dunnett’s Multiple Comparison Test (*P* < 0.05) was performed and showed no significant differences between the experiments and the control condition (V2 wild-type plants).

FIGURE S4 RT-PCR from recombinant PVX-infected *N. benthamiana* plants. Molecular analysis of plants infected with PVX-recombinant viruses expressing *V2* and *V2* mutants from BCTV. Total RNA was extracted from apical leaves of *N. benthamiana* plants infected with PVX-recombinant viruses expressing *V2* and *V2* mutated versions from BCTV. RT-PCR with specific primers for PVX was performed to quantify viral titer. As an internal control *N. benthamiana* gene *EF1* was used. Primers hybridizing at both sides of the MCS (*V2*) were also used to detect the retention of the geminiviral protein in the recombinant viruses.

FIGURE S5 Pearson’s correlation coefficient (R) for the co-localization of GFP and SUN1-tagRFP. Leaves were agroinfiltrated with a construct expressing the GFP-V2 fusion protein or the GFP-V2 mutants and the nuclear envelope marker AtSUN1–tagRFP and observed under the confocal microscope at 3 dpi. R was quantified and represented by box plots. Each box represents the mean values ± standard error (SE) from 6 to 9 nuclei.

FIGURE S6 Western blot from V2 protein (wild-type or mutated) fused to GFP. of samples of V2 mutants fused to GFP expressed in epidermal cells of *N. benthamiana* (Figure 5A). Leaves were agroinfiltrated with a construct expressing the 35S:GFP (GFP), 35S:GFP-V2 fusion protein or the 35S:GFP-V2 mutants (P1A, P1D, H1GG or H2GG). Samples were taken at 2 dpi (the same ones shown in Figure 5A) and total protein was extracted, loaded, resolved by 12% SDS-PAGE gel electrophoresis, and transferred by electroblotting onto a polyvinylidene diflouride membrane. Proteins were stained by Coomassie blue (CBB) and immunoblotted with anti-GFP mouse monoclonal antibody (α-GFP).

FIGURE S7 Infection of *N. benthamiana* plants with BCTV V2 mutants. Plants were agroinoculated with wild-type or V2 mutated BCTV clones. Number of symptomatic plants observed at 28 dpi. The asterisk indicates symptoms milder than the caused by the wild-type virus.
