# Supplementary figures and images for: Characterization of Curtovirus V2 Protein, a Functional Homolog of Begomovirus V2
Source: Front Plant Sci. 2020 Jun 19;11:835. doi: 10.3389/fpls.2020.00835 (PMC7318802; doi:10.3389/fpls.2020.00835)

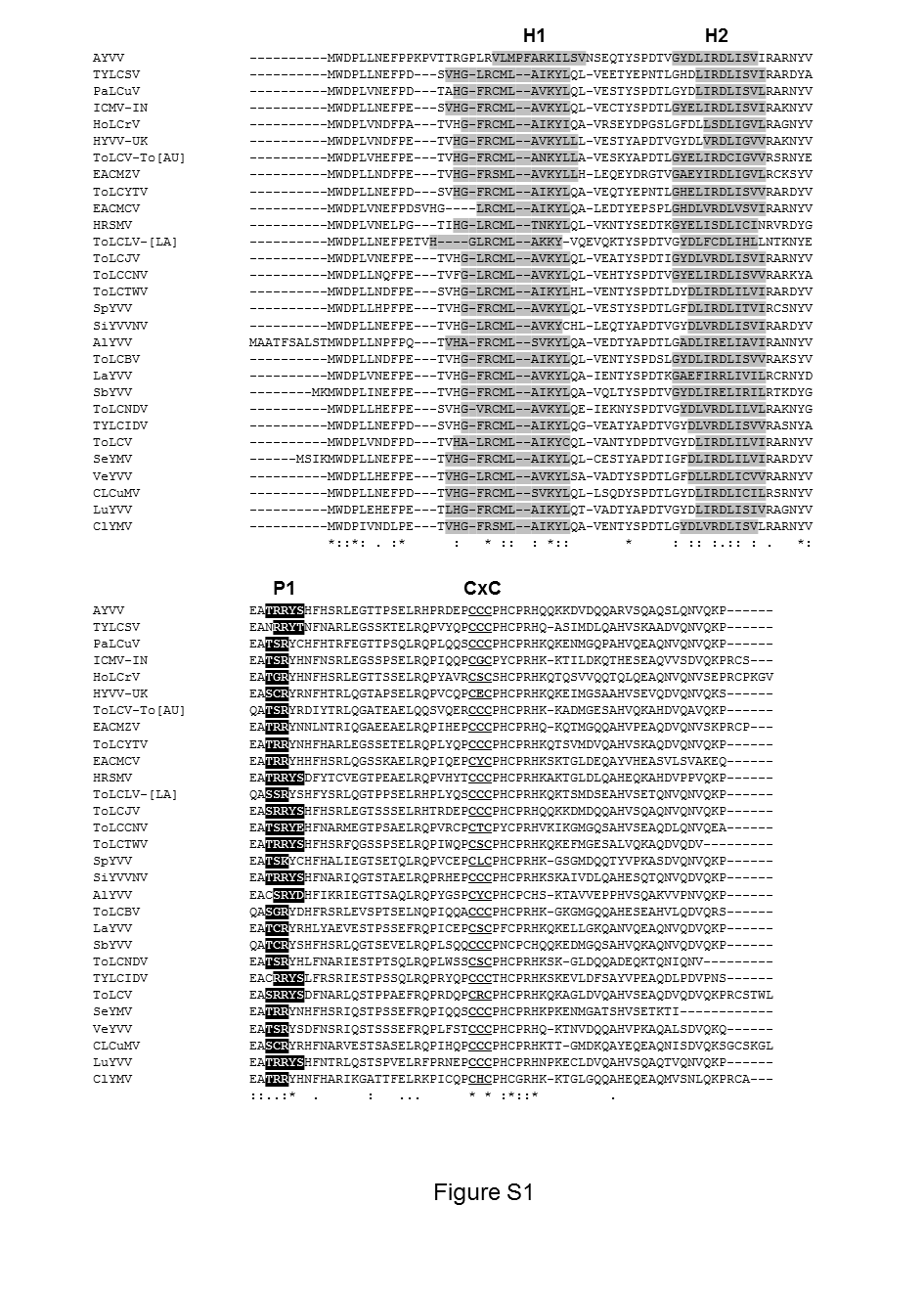

Supplement: FIGURE S1 — Alignment of the aminoacidic sequence of V2 from 29 begomovirus species: Ageratum yellow vein virus-China (AYVV/Gx; AJ495813), Tomato yellow leaf curl Sardinia virus (TYLCSaV; L27708), Papaya leaf curl Guandong virus (PaLCuGdV; AJ558122), Indian cassava mosaic virus (ICMV; AJ314739), Cotton leaf curl Gezira virus (CLCuGeV/Ca; AJ542539.1), Honeysuckle yellow vein virus-[UK] (HYVV-UK; AJ543429), Tomato leaf curl virus-Australia (ToLCV/To; S53251), East African cassava mosaic Zanzibar virus (EACMZV; AJ717559): Tomato leaf curl Mayotte virus (ToLCYTV; AJ620912), East African cassava mosaic Cameroon virus (EACMCV; AF112354); Hibiscus rosa-sinensis mosaic virus) (HRSMV; DQ022659), Tomato leaf curl Laos virus (ToLCLV; AF195782), Tomato leaf curl Java virus (ToLCJV; AB100304), Tomato leaf curl China virus (ToLCCNV; AJ811910), Tomato leaf curl Taiwan virus (ToLCTWV; DQ866127), Spilanthes yellow vein virus (SpYVV; DQ641694), Sida yellow vein Vietnam virus (SiYVVV; DQ641696), Alternanthera yellow vein virus (AlYVV; AM691015), Tomato leaf curl Bangalore virus (ToLCBV; AY428770), Lindernia anagallis yellow vein virus (LaYVV; AY795900), Siegesbeckia yellow vein virus (SgYVV; AM183224), Tomato leaf curl New Delhi virus (ToLCNDV; AY286316), Tomato yellow leaf curl Indonesia virus (TYLCIDV; AF189018), Tomato leaf curl Hsinchu virus (ToLCV; DQ866131), Senecio yellow mosaic virus (SeYMV; AJ876550), Vernonia yellow vein virus (VeYVV; AM182232), Cotton leaf curl Multan virus (CLCuMV; EF465535), Ludwigia yellow vein virus (LuYVV; AJ965539) and Clerodendron yellow mosaic virus (ClYMV; EF408037). Gaps (-) were introduced to optimize the alignment. The positions of the predicted putative phosphorilation motif P1 (protein kinase CK2/protein kinase C) are depicted in white letters inside black boxes. The CxC motif (Padidam et al., 1996; Zrachya et al., 2007) is underlined. The hydrophobic domains (H1 and H2) are shadowed in gray. [file Image_1.TIF]

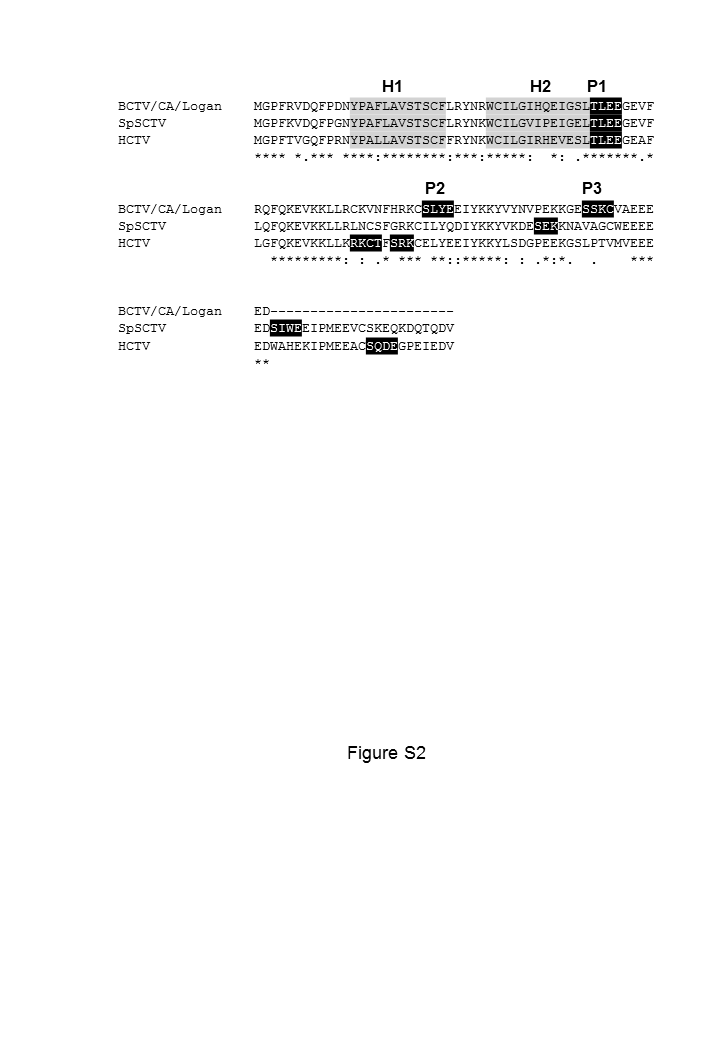

Supplement: FIGURE S2 — Alignment of the aminoacidic sequence of V2 from three curtovirus species: Beet curly top virus-California [Logan] (BCTV/CA/Logan; M24597), Spinach severe curly top virus (SpSCTV; GU734126) and Horseradish curly top virus (HCTV; U49907). The positions of the predicted putative phosphorylation motifs P1 (protein kinase CK2/protein kinase C), P2 (protein kinase CK2) and P3 (protein kinase C) are depicted in white letters inside black boxes. The hydrophobic domains (H1 and H2) are shadowed in gray. [file Image_2.TIF]

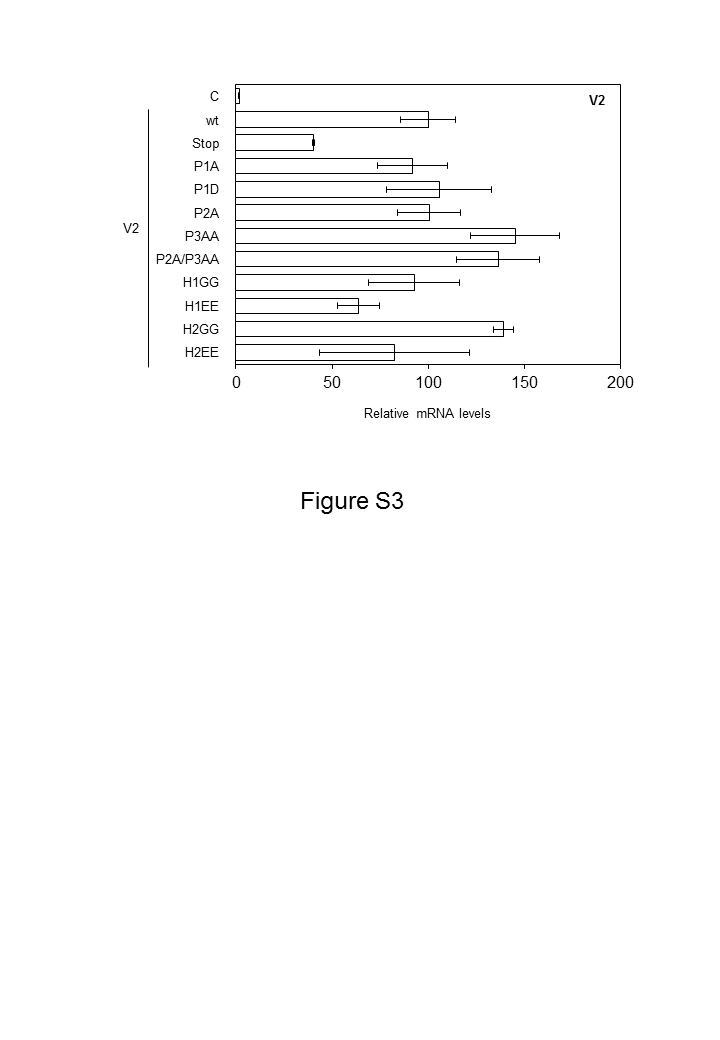

Supplement: FIGURE S3 — Relative V2 mRNA levels in N. benthamiana leaves. Leaves from N. benthamiana plants were infiltrated with a mixture of two A. tumefaciens cultures expressing GFP and the indicated version of V2 and relative V2 mRNA levels were measured by RT-qPCR in the infiltrated tissues at 1 dpi. Wild-type V2 protein (wt) and the empty vector (C) were used as a positive and negative controls, respectively. V2 transcript levels were normalized to EF1 and are presented as the relative amount of transcripts compared with the amount found in wild-type V2 (wt) samples (set to 100%). Bars represent the mean ± SD for three different pools from 2 to 3 leaves obtained from 3–4 plants each one. One Way ANOVA (Dunnett’s Multiple Comparison Test (P < 0.05) was performed and showed no significant differences between the experiments and the control condition (V2 wild-type plants). [file Image_3.TIF]

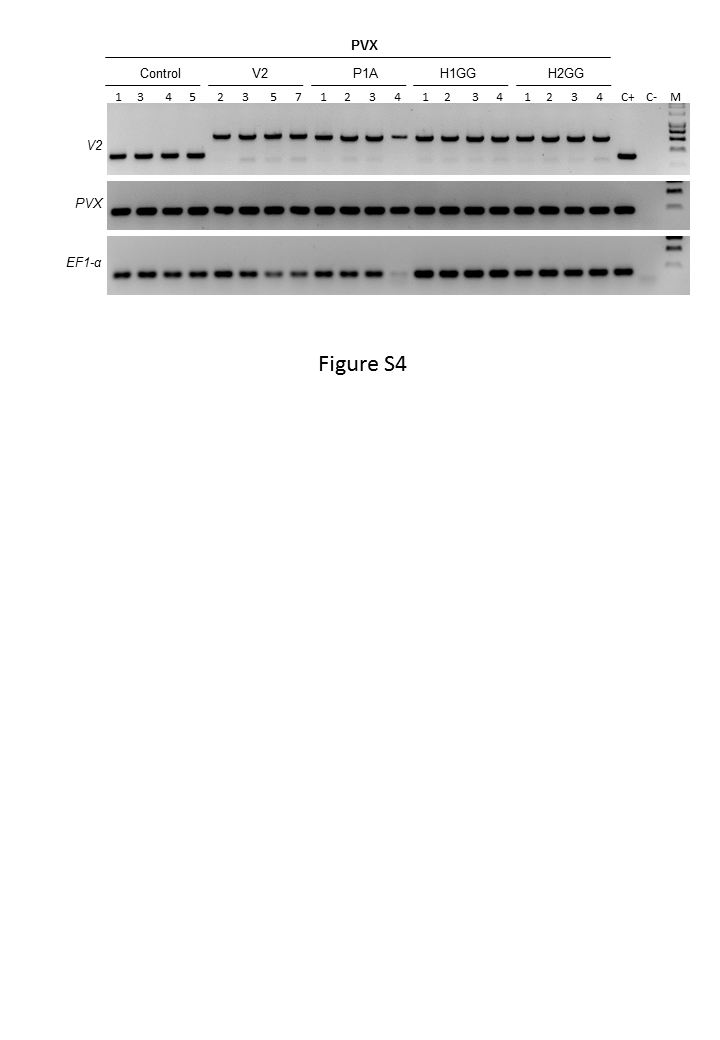

Supplement: FIGURE S4 — RT-PCR from recombinant PVX-infected N. benthamiana plants. Molecular analysis of plants infected with PVX-recombinant viruses expressing V2 and V2 mutants from BCTV. Total RNA was extracted from apical leaves of N. benthamiana plants infected with PVX-recombinant viruses expressing V2 and V2 mutated versions from BCTV. RT-PCR with specific primers for PVX was performed to quantify viral titer. As an internal control N. benthamiana gene EF1 was used. Primers hybridizing at both sides of the MCS (V2) were also used to detect the retention of the geminiviral protein in the recombinant viruses. [file Image_4.TIF]

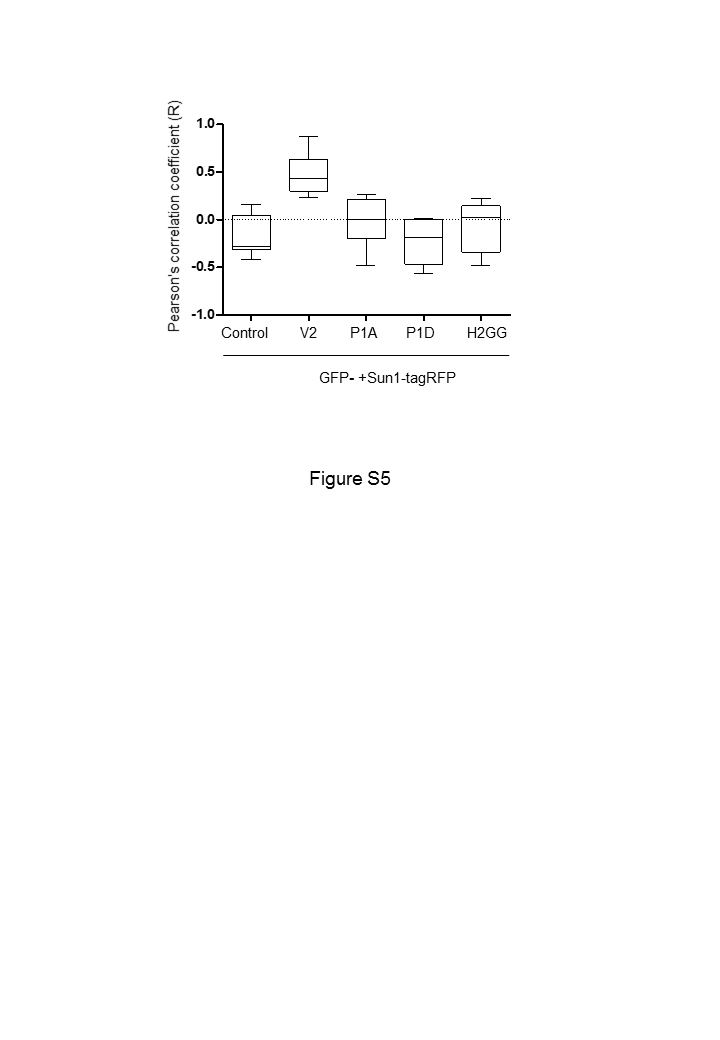

Supplement: FIGURE S5 — Pearson’s correlation coefficient (R) for the co-localization of GFP and SUN1-tagRFP. Leaves were agroinfiltrated with a construct expressing the GFP-V2 fusion protein or the GFP-V2 mutants and the nuclear envelope marker AtSUN1–tagRFP and observed under the confocal microscope at 3 dpi. R was quantified and represented by box plots. Each box represents the mean values ± standard error (SE) from 6 to 9 nuclei. [file Image_5.TIF]

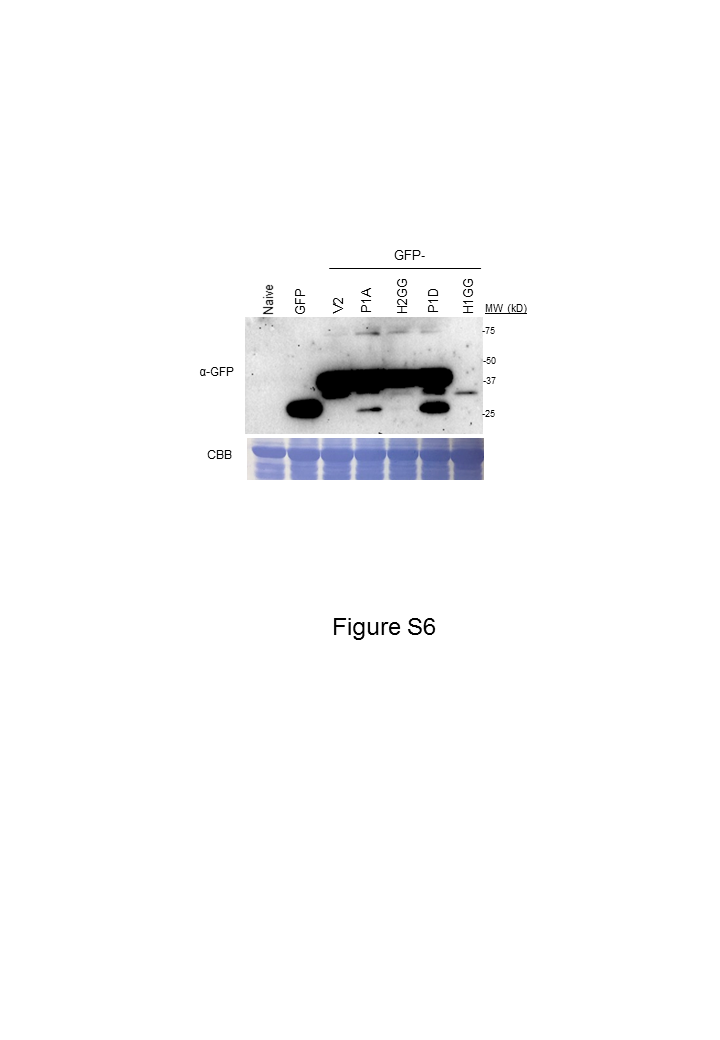

Supplement: FIGURE S6 — Western blot from V2 protein (wild-type or mutated) fused to GFP. of samples of V2 mutants fused to GFP expressed in epidermal cells of N. benthamiana (Figure 5A). Leaves were agroinfiltrated with a construct expressing the 35S:GFP (GFP), 35S:GFP-V2 fusion protein or the 35S:GFP-V2 mutants (P1A, P1D, H1GG or H2GG). Samples were taken at 2 dpi (the same ones shown in Figure 5A) and total protein was extracted, loaded, resolved by 12% SDS-PAGE gel electrophoresis, and transferred by electroblotting onto a polyvinylidene diflouride membrane. Proteins were stained by Coomassie blue (CBB) and immunoblotted with anti-GFP mouse monoclonal antibody (α-GFP). [file Image_6.TIF]

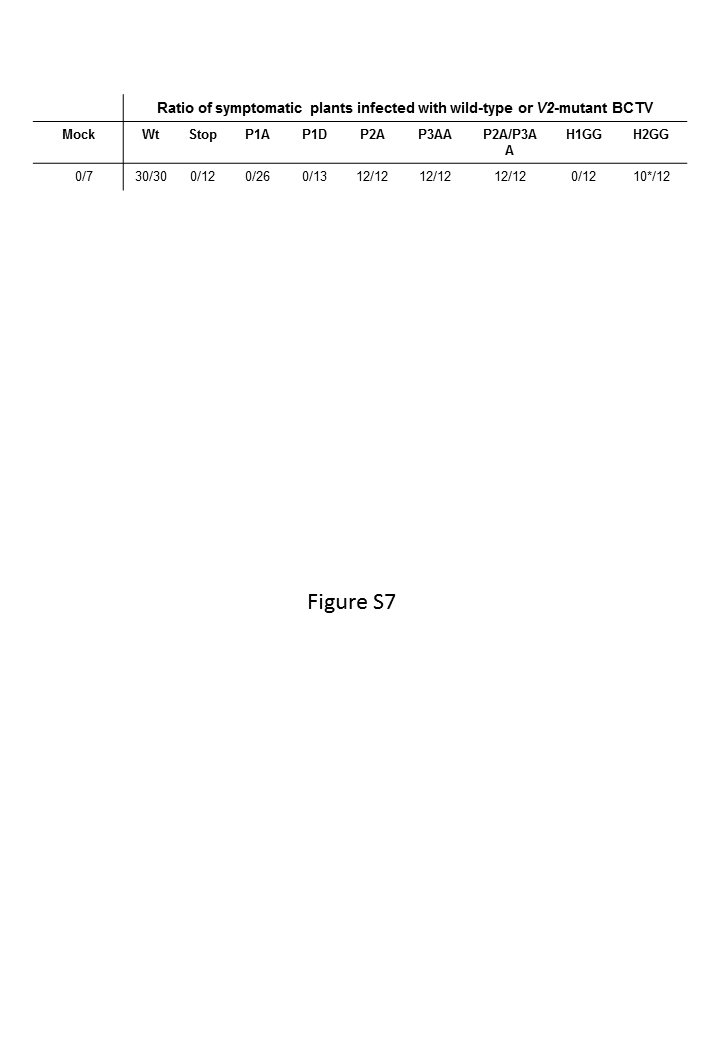

Supplement: FIGURE S7 — Infection of N. benthamiana plants with BCTV V2 mutants. Plants were agroinoculated with wild-type or V2 mutated BCTV clones. Number of symptomatic plants observed at 28 dpi. The asterisk indicates symptoms milder than the caused by the wild-type virus. [file Image_7.TIF]
