# Supplementary material for: Characterization of Curtovirus V2 Protein, a Functional Homolog of Begomovirus V2
Source: Front Plant Sci. 2020 Jun 19;11:835. doi: 10.3389/fpls.2020.00835 (PMC7318802; doi:10.3389/fpls.2020.00835)
Supplement: Supplementary file 8 [file Table_1.docx]

| **Plasmid** | **Vector backbone** | **Fragment origin** | **Primers or fragment** | **Cloning site** | **Notes** |
| --- | --- | --- | --- | --- | --- |
|  | | | | | |
| **Subcloning** | | | | | |
| pV2BC | - - 1. pBSSKII+(a) | pBIN 1.2 (b) | - - 1. upV2BC/lowV2BC | - - 1. *EcoR*V | Described in Luna et al., 2017  (doi: 10.1099/jgv.0.000933) |
| pV2P1A | '' | pV2BC | V2P1A (Table S3) | *EcoR*V | BCTV V2_1-102_ T43A |
| pV2P2A | '' | “ | - - 1. V2P2A(Table S3) | - - 1. *EcoR*V | BCTV V2_1-102_ S72A |
| pV2P3AA | '' | “ | V2P3AA (Table S3) | - - 1. *EcoR*V | BCTV V2_1-102_ S92A/S93A |
| pV2H1GG | '' | “ | V2H1GG (Table S3) | *EcoR*V | BCTV V2_1-102_ L17G/V19G |
| pV2H1EE | '' | “ | V2H1EE (Table S3) | *EcoR*V | BCTV V2_1-102_ L17E/V19E |
| pV2H2GG | - - 1. '' | - - 1. “ | - - 1. V2H2GG (Table S3) | - - 1. *EcoR*V | BCTV V2_1-102_ I32G/I35G |
| pV2H2EE | '' | “ | V2H2EE (Table S3) | *EcoR*V | BCTV V2_1-102_ I32E/I35E |
| pV2stop | '' | “ | V2Stop (Table S3) | *EcoR*V | BCTV V2 _1-17_ stop |
| pENTR-V2BC | pENTRD-TOPO (c) | pV2BC | V2-attB1 Fw/ V2-attB1 Low and attB1/2 ADAPTERs | *-* | BCTV V2_1-102_ |
|  |  |  |  |  |  |
| pENTR-V2P1A | '' | pV2P1A | V2-attB1 Fw/ V2-attB1 Low and attB1/2 ADAPTERs | *-* | BCTV V2_1-102_ T43A |
| pENTR-V2H1GG | '' | pV2H1GG | V2-attB1 Fw/ V2-attB1 Low and attB1/2 ADAPTERs | *-* | BCTV V2_1-102_ L17G/V19G |
| pENTR-V2H2GG | '' | pV2H2GG | V2-attB1 Fw/ V2-attB1 Low and attB1/2 ADAPTERs | *-* | BCTV V2_1-102_ I32G/I35G |
| pg0.2BCTV | pGreen-S (d) | 0.2 genome of BCTV | pBIN1.2 *Sac*I-*Sal*I (b) | *Sac*I-*Sal*I | Subcloning for the construction of an infective clone for BCTV |
|  | | | | | |
| **Plant binary expression vectors** | | | | | |
| pBIV2BC | pBINX1 (e) | pV2BC | pV2BC *BamH*I-*Sal*I | *BamH*I-*Sal*I | Described in Luna et al., 2017  (doi: 10.1099/jgv.0.000933) |
| pBIV2P1A | - - 1. '' | pV2P1A | pV2P1A *BamH*I-*Sal*I | - - - 1. *BamH*I-*Sal*I | BCTV V2_1-102_ T43A |
| pBIV2P1D | pGWB2 (f) | pENTR-V2P1D(g) | - | *-* | BCTV V2_1-102_ T43D |
| pBIV2P2A | pBINX1 (e) | pV2P2A | pV2P2A *BamH*I-*Sal*I | *BamH*I-*Sal*I | BCTV V2_1-102_ S72A |
| pBIV2P3AA | '' | pV2P3AA | pV2P3AA *BamH*I-*Sal*I | *BamH*I-*Sal*I | BCTV V2_1-102_ S92A/S93A |
| pBIV2H1GG | '' | pV2H1GG | pV2H1GG *BamH*I-*Sal*I | *BamH*I-*Sal*I | BCTV V2_1-102_ L17G/V19G |
| pBIV2H1EE | '' | pV2H1EE | pV2H1EE *BamH*I-*Sal*I | *BamH*I-*Sal*I | BCTV V2_1-102_ L17E/V19E |
| pBIV2H2GG | '' | pV2H2GG | pV2H2GG *BamH*I-*Sal*I | *BamH*I-*Sal*I | BCTV V2_1-102_ I32G/I35G |
| pBIV2H2EE | '' | pV2H2EE | pV2H2EE *BamH*I-*Sal*I | *BamH*I-*Sal*I | BCTV V2_1-102_ I32E/I35E |
| pBIV2stop | '' | pV2stop | pV2stop *BamH*I-*Sal*I | *BamH*I-*Sal*I | BCTV V2 _1-17_ stop |
| pBIV2P2A/P3AA | '' | pV2P2A/P3AA(g) | pV2P2A/P3AA *BamH*I-*Sal*I | *BamH*I-*Sal*I | BCTV V2_1-102_ S72A/S92A/S93A |
|  |  |  |  |  |  |
|  |  |  |  |  |  |
|  |  |  |  |  |  |
| **Plasmid** | **Vector backbone** | **Fragment origin** | **Primers or fragment** | **Cloning site** | **Notes** |
|  |  |  |  |  |  |
| pBI-GFPV2P1A | pGWB6 (f) | pENTR-V2P1A | - | *-* | GFP-BCTV V2_1-102_ T43A |
| pBI-GFPV2P1D | '' | pENTR-V2P1D | - | *-* | GFP-BCTV V2_1-102_ T43D |
| pBI-GFPV2H1GG | '' | pENTR-V2H1GG | - | *-* | GFP-BCTV V2_1-102_ L17G/V19G |
| pBI-GFPV2H2GG | pGWB6 (f) | pENTR-V2H2GG | - | *-* | GFP-BCTV V2_1-102_ I32G/I35G |
|  |  |  |  |  |  |
| **PVX vectors** | | | | | |
| PVX-V2BC | PGR107 (h) | pV2BC | pV2BC *Sma*I-*Cla*I |  |  |
| PVX-V2P1A | '' | pV2P1A | pV2P1A *Sma*I-*Cla*I | *Sma*I-*ClaI* | BCTV V2_1-102_ T43A |
| PVX-V2H1GG | '' | pV2H1GG | pV2H1GG *Sma*I-*Cla*I | *SmaI-ClaI* | BCTV V2_1-102_ L17G/V19G |
| PVX-V2H2GG | '' | pV2H2GG | pV2H2GG *Sma*I-*Cla*I | *Sma*I-*Cla*I | BCTV V2_1-102_ I32G/I35G |
|  |  |  |  |  |  |
| **Infectious clones** | | | | | |
| - - 1. pg1.2BCTV | pg0.2BCTV | pBIN1.2 (b) | - - 1. pBIN1.2 *Sal*I | - - 1. *Sal*I | BCTV Wt |
| pg1.2BC-V2P1A | See table IV | See table IV | See table IV | See table IV | BCTV V2P1A |
| pg1.2BC-V2P1D | '' | '' | '' | '' | BCTV V2P1D |
| pg1.2BC-V2P2A | '' | '' | '' | '' | BCTV V2P2A |
| pg1.2BC-V2P3AA | '' | '' | '' | '' | BCTV V2P3AA |
| pg1.2BC-V2P2A/P3AA | '' | '' | '' | '' | BCTV V2P2A/P3AA |
| pg1.2BC-V2H1GG | '' | '' | '' | '' | BCTV V2H1GG |
| pg1.2BC-V2H2GG | '' | '' | '' | '' | BCTV V2H2GG |
| pg1.2BC-V2stop | - - 1. '' | '' | - - 1. '' | - - 1. '' | BCTV V2Stop |
|  |  |  |  |  |  |
|  |  |  |  |  |  |

**Table S1.** Plasmids generated in this work. All plasmid names that contain a BCTV ORF are ended in BC or BCTV. The backbone, fragment origin, fragment or primer used, cloning site, and notes are shown. Plasmids origins are: (a): Stratagene; (b): (Briddon et al., 1989 doi: 10.1016/0042-6822(89)90205-5); (c): Invitrogen; (d): To yield pGreen-S, the binary plasmid pGreenII 0229 (Hellens et al., 2000 doi: 10.1023/a:1006496308160) was digested with SalI to release a 0.81 Kb fragment, and re-ligated. (e): (Sanchez-Duran et al., 2011 doi: 10.1128/JVI.02566-10); (f): (Nakagawa et al., 2007 doi: 10.1263/jbb.104.34; (g) pENTR-V2P1D and pV2P2A/P3AA were generated using QuikChange Lightning Site-Directed Mutagenesis kit (Stratagene, Agilent biotechnologies) following the manufacturer's instructions. Primers used were lowV2P1D-upV2P1D or upV2P2-lowV2P2 and the plasmids pENTR-V2BC or pV2P3AA as templates respectively.; (h): (Jones et al., 1999 doi: 10.1105/tpc.11.12.2291).
