# Supplementary material for: Characterization of Curtovirus V2 Protein, a Functional Homolog of Begomovirus V2
Source: Front Plant Sci. 2020 Jun 19;11:835. doi: 10.3389/fpls.2020.00835 (PMC7318802; doi:10.3389/fpls.2020.00835)
Supplement: Supplementary file 9 [file Table_2.docx]

| **Primer** | **Oligonucleotide sequence 5’ -> 3’** | **Use** | |  |
| --- | --- | --- | --- | --- |
| LowV2BC | GTACTAGTCCTCCTCTTCTTCG | Subcloning | | |
| UpV2BC | TTTATGGGACCTTTCAGAGTGG | Subcloning | | |
| V2-attB1 Fw | GACGAAAGACCTCGCCTTCT | Cloning in Gateway | | |
| V2-attB2 Rv | GAAAGCTGGGTcCTAGTCCTCCTCTTCTTCGGCC | Cloning in Gateway | | |
| attB1 ADAPTER | GGGGACAAGTTTGTACAAAAAAGCAGGCT | Cloning in Gateway | | |
| attB2 ADAPTER | GGGGACCACTTTGTACAAGAAAGCTGGGT | Cloning in Gateway | | |
| LowPBSK | CAGCTGGCACGACAGGTTTCCCGAC | V2 mutagenesis | | |
| UpPBSK | CGGTGCGGGCCTCTTCGCTATTAC | V2 mutagenesis | | |
| LowV2P1A | CGCCTTCTTCTAGGGCCAGAGACCCTATCTC | V2 mutagenesis | | |
| UpV2P1A | GAGATAGGGTCTCTGGCCCTAGAAGAAGGCG | V2 mutagenesis | | |
| LowV2P1D | CTCGCCTTCTTCTAGgtcCAGAGACCCTATCTCTTGATG | V2 mutagenesis | | |
| UpV2P1D | CATCAAGAGATAGGGTCTCTGgacCTAGAAGAAGGCGAG | V2 mutagenesis | | |
| LowV2P2A | GTATATTTCCTCATACAACGCACACTTCCTATG | V2 mutagenesis | | |
| UpV2P2A | CATAGGAAGTGTGCGTTGTATGAGGAAATATAC | V2 mutagenesis | | |
| LowV2P3AA | CGGCCACGCACTTTGCGGCTTCACCTTTCTTTTC | V2 mutagenesis | | |
| UpV2P3AA | GAAAAGAAAGGTGAAGCCGCAAAGTGCGTGGCCG | V2 mutagenesis | | |
| LowV2H1GG | CAACTGGTCGATCCTGCTCCAAAGGCTGGATAATTG | V2 mutagenesis | | |
| UpV2H1GG | CAATTATCCAGCCTTTGGAGCAGGATCGACCAGTTG | V2 mutagenesis | | |
| LowV2H1EE | CAACTGGTCGATTCTGCTTCAAAGGCTGGATAATTG | V2 mutagenesis | | |
| UpV2H1EE | CAATTATCCAGCCTTTGAAGCAGAATCGACCAGTTG | V2 mutagenesis | | |
| LowV2H2GG | CTATCTCTTGATGGCCACCTAGTCCACACCACCTGTTG | V2 mutagenesis | | |
| UpV2H2GG | CAACAGGTGGTGTGGACTAGGTGGCCATCAAGAGATAG | V2 mutagenesis | | |
| LowV2H2EE | CTATCTCTTGATGCTCACCTAGTTCACACCACCTGTTG | V2 mutagenesis | | |
| UpV2H2EE | AGGTGGTGTGAACTAGGTGAGCATCAAGAGATAGGGTC | V2 mutagenesis | | |
| LowV2stop | GATAATTGTCTGGAAATTGATATCCACTCTGAAAGG | V2 mutagenesis | | |
| UpV2stop | CCTTTCAGAGTGGATATCAATTTCCAGACAATTATC | V2 mutagenesis | | |
| LowV2BC-qRT(a) | GACGAAAGACCTCGCCTTCT | RT-qPCR (V2 mRNA amplification) | | |
| UpV2BC-qRT | ATGGGACCTTTCAGAGTGGA | RT-qPCR (V2 mRNA amplification) | | |
| LowGFP-qRT(a) | GATCCTGTTGACGAGGGTGT | RT-qPCR (GFP mRNA amplification) |  |  |
| UpGFP-qRT | GAGGGATACGTGCAGGAGAG | RT-qPCR (GFP mRNA amplification) |  |  |
| LowEF-1a NB (b) | AGCTTCGTGGTGCATCTC | RT-qPCR (EF1alfa mRNA amplification) |  |  |
| UpEF-1a NB | GATTGGTGGTATTGGAACTGTC | RT-qPCR (EF1alfa mRNA amplification) |  |  |
| LowBCTV-qRT(a) | CTACACGAAGATGGGCAACCT | qPCR (BCTV amplification) |  |  |
| UpBCTV-qRT | TGACGTCGGAGCTGGATTTAG | qPCR (BCTV amplification) |  |  |
| Low 3´-PVX (c) | TCTAGGCTGGCAAAGTCGTT | RT-PCR (PVX amplification) |  |  |
| Up 3´-PVX | ATTCGCTGCATTCGACTTCT | RT-PCR (PVX amplification) |  |  |
| PVX-MCS forward (d) | AATCATAGCAGTCATTAGCACTTCC | RT-PCR (V2 amplification) |  |  |
| PVX-MCS reverse | TTTGTGGTAGTTGAGGTAGTTGACC | RT-PCR (V2 amplification) |  |  |
| Actin Fw(e) | GGCAAGTCATCACGATTGG | qPCR (actin amplification) |  |  |
| Actin Rv | CAGCTTCCATTCCCACAAAC | qPCR (actin amplification) |  |  |
| 25SrRNA UNIV (-) (f) | CCGAAGTTACGGATCCATTT | qPCR (ITS amplification) |  |  |
| 25SrRNA UNIV (+) | ATAACCGCATCAGGTCTCCA | qPCR (ITS amplification) |  |  |

**Table S2. Primers used in this work:** (a): (Luna et al., 2017 doi: 10.1099/jgv.0.000933); (b): (Rotenberg et al., 2006 doi: 10.1016/j.jviromet.2006.07.017); (c): Designed to amplify a 168 bp fragment in region 3´of PVX genome. Acc.: AY297842.1 (d): (Cañizares et al., 2008 doi: 10.1016/j.virol.2008.06.020); (e) (Ishikawa et al., 2010 doi: 10.1104/pp.110.153569); (f): (Mason et al., 2008 doi: 10.1016/j.jviromet.2007.09.015).
