# Supplementary material for: Characterization of Curtovirus V2 Protein, a Functional Homolog of Begomovirus V2
Source: Front Plant Sci. 2020 Jun 19;11:835. doi: 10.3389/fpls.2020.00835 (PMC7318802; doi:10.3389/fpls.2020.00835)
Supplement: Supplementary file 10 [file Table_3.docx]

| **Mutant name** | **Mutation in V2** | **Primers for upstream fragment** | **Primers for downstream fragment** |
| --- | --- | --- | --- |
| **V2P1A** | T43A | upPBSK-lowV2P1A | upV2P1A-lowPBSK |
| **V2P2A** | S72A | upPBSK-lowV2P2A | upV2P2A-lowPBSK |
| **V2P3AA** | S92A/S93A | upPBSK-lowV2P3AA | upV2P3AA-lowPBSK |
| **V2H1GG** | L17G/V19G | upPBSK-lowV2H1GG | upV2H1GG-lowPBSK |
| **V2H1EE** | L17E/V19E | upPBSK-lowV2H1EE | upV2H1EE-lowPBSK |
| **V2H2GG** | I32G/I35G | upPBSK-lowV2H2GG | upV2H2GG-lowPBSK |
| **V2H2EE** | I32E/I35E | upPBSK-lowV2H2EE | upV2H2EE-lowPBSK |
| **V2stop** | Stop signal (*) | upPBSK-lowV2stop | upV2stop-lowPBSK |
|  |  |  |  |

**Table S3. Generation of mutated BCTV V2 ORFs:** primers used for the generation of BCTV V2 mutants by two-sided splicing by over-lap extension (Ho et al., 1989 doi: 10.1016/0378-1119(89)90358-2). For each mutant, two PCR amplifications were performed using pV2BC as a template to generate fragments flanking the codons to be substituted (upstream and downstream) with the primers described here. Once the two fragments for each mutant were obtained, these were used as template DNA for a new PCR amplification without primers and a final amplification with external primers. (*): An AT dinucleotide was inserted, after nucleotide 395 in BCTV genome (Accession number: M24597), producing a premature stop signal and a truncated V2 protein of 17 aa.
