# Supplementary material for: Characterization of Curtovirus V2 Protein, a Functional Homolog of Begomovirus V2
Source: Front Plant Sci. 2020 Jun 19;11:835. doi: 10.3389/fpls.2020.00835 (PMC7318802; doi:10.3389/fpls.2020.00835)
Supplement: Supplementary file 11 [file Table_4.PDF]

Mutant name	V2 mutation	Template used	Primers	Resultant binary vector
**BCTV V2P1A**	T43A	pg1.2BCTV	upV2P1A-lowV2P1A	pg1.2BC-V2P1A
**BCTV V2P1D**	T43D	pg1.2BCTV	upV2P1D-lowV2P1D	pg1.2BC-V2P1D
**BCTV V2P2A**	S72A	pg1.2BCTV	upV2P2A-lowV2P2A	pg1.2BC-V2P2A
**BCTV V2P3AA**	S92A/S93A	pg1.2BCTV	upV2P3AA-lowV2P3AA	pg1.2BC-V2P3AA
**BCTV V2P2A/P3AA**	S72A/ S92A/S93A	pg1.2BC-V2P3AA	upV2P2A-lowV2P2A	pg1.2BC-V2P2-1/P3-1
**BCTV V2H1GG**	L17G/V19G	pg1.2BCTV	upV2H1GG-lowV2H1GG	pg1.2BC-V2H1GG
**BCTV V2H2GG**	I32G/I35G	pg1.2BCTV	upV2H2GG-lowV2H2GG	pg1.2BC-V2H2GG
**BCTV V2stop**	Stop signal (*)	pg1.2BCTV	upV2stop-lowV2stop	pg1.2BC-V2stop

Table S4. Generation of point mutation in V2 in the infective BCTV clone pg1.2BCTV: Point mutations in V2 were generated in pg1.2BCTV by the *in vitro* site-directed mutagenesis with QuikChange Lightning Site-Directed Mutagenesis kit (Stratagene, Agilent biotechnologies). Primers and template used are indicated. (*): An AT dinucleotide was inserted after nucleotide 395 in BCTV genome, producing a premature stop signal and a truncated V2 protein of 17 aa (V2stop; MGPFRVDINFQTIIQPF*).
